# The study of a barley epigenetic regulator, *HvDME,* in seed development and under drought

**DOI:** 10.1186/1471-2229-13-172

**Published:** 2013-10-31

**Authors:** Aliki Kapazoglou, Vicky Drosou, Anagnostis Argiriou, Athanasios S Tsaftaris

**Affiliations:** 1 Institute of Applied Biosciences (INAB), CERTH, Thermi-Thessaloniki GR-57001, Greece; 2 Department of Genetics and Plant Breeding, Aristotle University of Thessaloniki, Thessaloniki GR-54124, Greece

**Keywords:** DEMETER (DME), DNA glycosylase, Epigenetic regulation, Chromatin, DNA methylation, DNA demethylation, Endosperm, Seed development, Drought, Barley, Retrotransposon, Intronic miRNA

## Abstract

**Background:**

Epigenetic factors such as DNA methylation and histone modifications regulate a wide range of processes in plant development. Cytosine methylation and demethylation exist in a dynamic balance and have been associated with gene silencing or activation, respectively. In Arabidopsis, cytosine demethylation is achieved by specific DNA glycosylases, including AtDME (DEMETER) and AtROS1 (REPRESSOR OF SILENCING1), which have been shown to play important roles in seed development. Nevertheless, studies on monocot DNA glycosylases are limited. Here we present the study of a *DME* homologue from barley *(HvDME)*, an agronomically important cereal crop, during seed development and in response to conditions of drought.

**Results:**

An *HvDME* gene, identified in GenBank, was found to encode a protein with all the characteristic modules of DME-family DNA glycosylase proteins. Phylogenetic analysis revealed a high degree of homology to other monocot DME glycosylases, and sequence divergence from the ROS1, DML2 and DML3 orthologues. The *HvDME* gene contains the 5′ and 3′ Long Terminal Repeats (LTR) of a *Copia* retrotransposon element within the 3′ downstream region. *HvDME* transcripts were shown to be present both in vegetative and reproductive tissues and accumulated differentially in different seed developmental stages and in two different cultivars with varying seed size. Additionally, remarkable induction of *HvDME* was evidenced in response to drought treatment in a drought-tolerant barley cultivar. Moreover, variable degrees of DNA methylation in specific regions of the *HvDME* promoter and gene body were detected in two different cultivars.

**Conclusion:**

A gene encoding a DNA glycosylase closely related to cereal DME glycosylases was characterized in barley. Expression analysis during seed development and under dehydration conditions suggested a role for *HvDME* in endosperm development, seed maturation, and in response to drought. Furthermore, differential DNA methylation patterns within the gene in two different cultivars suggested epigenetic regulation of *HvDME*. The study of a barley *DME* gene will contribute to our understanding of epigenetic mechanisms operating during seed development and stress response in agronomically important cereal crops.

## Background

Epigenetic regulation during plant development and in response to environmental conditions is attained by DNA methylation, histone modifications, small interfering RNAs (siRNAs) and long non-coding RNAs (Long ncRNAs), leading to changes in chromatin structure. Open and closed chromatin states are associated with gene activation and gene silencing, respectively, and govern proper onset of gene expression programmes during different developmental processes and in response to changing environmental conditions [1-3].

A dynamic interplay between DNA methylation and demethylation accomplished through specific enzymes, is critical for proper cellular regulation during plant development [4,5]. Even though DNA methylation is a relatively stable epigenetic mark, it is subject to passive or active demethylation during the development of an organism [6]. Passive demethylation can occur when methylated cytosines are replaced by non-modified cytosines during DNA replication. Active DNA demethylation is achieved by specific DNA glycosylases through the base-excision-repair (BER) pathway by hydrolysis of the N-glycosidic bond between the ribose and the base [5,7,8]. Recent studies in animals have implicated another mechanism for DNA demethylation initiating with the hydroxylation of 5 methyl-cytosine by TET1 methylcytosine dioxygenase followed by the BER pathway that leads to DNA demethylation [9-11]. Such a mechanism is not found in plants, thus far. In plants demethylation by the BER pathway is operated by DNA glycosylases that excise 5 methylcytosine from the DNA sugar backbone and cleave the backbone at the abasic site [6]. In Arabidopsis, four such DNA glycosylase have been described, DEMETER (DME), REPRESSOR OF SILENCING (ROS1), DEMETER LIKE2 (DML2) and DEMETER LIKE3 (DML3), also called DME-family DNA glycosylases. AtDME and AtROS1 are bifunctional DNA glycosylases/AP-lyases that excise 5-methylcytosine and subsequently cleave the ribose-base phosphodiester bond whereas the resulting gap is filled by a DNA polymerase and the repair process is completed by a DNA ligase [12-15].

DME-family DNA glycosylases (DME, ROS1, DML2, DML3) have both common and different structural features as compared to typical DNA glycosylases. The glycosylase domain of DME-family proteins harbours the conserved helix–hairpin–helix (HhH) motif and a glycine/proline-rich region followed by a conserved aspartic acid (GPD) also found in human 8-oxoguanine DNA glycosylase (hOGG1), *Escherichia coli* adenine DNA glycosylase (MutY), and endonuclease III (Endo III) [16-18]. Moreover, similar to MutY and Endo III, DME-family DNA glycosylases contain four conserved cysteine residues flanking the DNA glycosylase domain that may function to hold a [4Fe-4S] cluster in place. Unlike other members of the HhH DNA glycosylase superfamily, DME-family members contain two additional conserved domains (domain A and domain B) flanking the central glycosylase domain [18]. Mutagenesis analysis of AtDME has revealed that the conserved DNA glycosylase domain and flanking domains A, and B, are necessary and sufficient for DME enzymatic activity [18].

Initial reports had implicated *AtDME* in demethylating genes of the female gametophyte involved in endosperm development whereas *AtROS1*, *AtDML2* and *AtDML3* were found expressed in vegetative tissues targeting transposons, repetitive elements and small RNA-generating loci [19-21]. *AtDME* was originally characterized as an epigenetic regulator required for maternal allelelic expression of the *MEDEA* (*MEA*) gene, encoding a H3K27 methylatransferase, in the central cell and endosperm [12,22,23]. In Arabidopsis, proper embryo and endosperm development depends on the expression of the maternal allele of the Polycomb group Polycomb Repressive complex (PRC2) encoding genes: *MEA, FERTILIZATION INDEPENDENT ENDOSPERM (FIE)*, and *FERTILIZATION INDEPENDENT SEED2 (FIS2*) [22-24]. MEA, FIE and FIS2 play a role in preventing the onset of central cell proliferation by repressing the expression of target genes, among them the Type-I MADS-box gene PHERES1 (PHE1) [23,25,26].

The first reports on global DNA methylation profiling of endosperm and embryo genomes demonstrated wide-spread reduction of DNA methylation in the endosperm, particularly at regions corresponding to TE and small RNAs [27,28]. These were largely due to *AtDME* action in the central cell, the progenitor of the endosperm, which develops after fusion of the central cell with one of the sperm cells of the pollen. Though *AtDME* action is restricted to the central cell, global demethylation persists in the developing endosperm post-fertilization. It was proposed that imprinted genes are not specific sequences targeted for demethylation but rather the result of a universal process carried out primarily through the action of DME that reconfigures DNA methylation of the entire maternal genome in the endosperm [28]. Recently it was demonstrated that *AtDME* is responsible for all of the active DNA demethylation taking place in the central cell and it preferentially targets small, AT-rich, and nucleosome-depleted euchromatic transposable elements [29]. AtDME also demethylates similar sequences in the vegetative cell of the male gametophyte and suppression of *AtDME* in the vegetative cell causes reduced small RNA–directed DNA methylation of transposons in its companion sperm cell [29-31].

Unlike the extensive investigations in Arabidopsis, studies on DNA glycosylases in monocots are limited. DNA demethylation was shown to result in the activation of a wide range of protein coding genes as well as transposable elements in rice endosperm. Interestingly, knockout mutants of a rice *ROS1 *gene which demethylates retrotransposn *Tos17* led to wrinkled seeds as compared to wild type plants, suggesting that rice *ROS1* is involved in seed development [32]. Likewise, a null mutation of rice *ROS1a* leads to abnormal early endosperm development and nonviable seed [33]. Finally, extensive efforts to understand and treat gluten-intolerance and celiac disease in population groups have led to the isolation and genetic engineering of wheat and barley *DME* homologues (among other genes) [34-39]. Importantly, downregulation of wheat *DME* resulted in decreased expression of endosperm prolamins, a powerful immunoreactant in celiac disease patients [39].

During the past several years our group has studied genes encoding epigenetic regulators and their putative targets, during seed development and in response to stress in barley, an agronomically important cereal crop [40-48]. In this study we have extended our exploration of epigenetic regulation in barley, by investigating the possible role of an *HvDME* gene, in seed development and in response to drought stress in different barley cultivars.

## Methods

### Plant material

Seeds for commercial barley cultivars, Caresse, Kos, Ippolytos and Demetra differing in seed size, weight and drought tolerance, were kindly provided by the Cereal Institute at the National Agricultural Research Foundation of Greece (http://www.cerealinstitute.gr) and were the source of total RNA and genomic DNA. For Caresse the weight of 1000 grains is 50–55 gr and 98% of seeds have diameter longer than 2.5 mm, for Kos the weight of 1000 grains is 36–40 gr and 75% of seeds have diameter longer than 2.5 mm, whereas for Ippolytos, seeds weight 25–31 gr per 1000 grains and only 35–45% of seeds have diameter longer than 2.5 mm. Caresse has facultative growth and is characterized by intermediate tolerance to abiotic stress/drought whereas Demetra is also a facultative type cultivar, drought-tolerant and very adaptable to a variety of soil-climatic conditions. The weight per 1,000 grains is 38–44 g and 70% of seeds have diameter longer than 2.5 mm (http://www.cerealinstitute.gr).

### Drought experiment

An open hydroponic-type arrangement was used for the experimental setup consisting of 6 pots from each cultivar (Caresse and Demetra), which were constantly irrigated with tap water (pH 6–7). Three seedlings were grown inside each pot. Seedlings were allowed to grow for up to 7 days, at which time 3 pots were removed from the hydroponic setup and placed into separate dry plates. These were water-withheld for a total of 10 days. The other 3 pots were used as controls and were kept in well-watered conditions. Aerial parts from each pot were pulled together so each biological repeat is represented by 9 plants. Two biological replicates were conducted. The aerial parts of seedlings were harvested the 3rd and 10th day and were stored in −80°C until further use.

### RNA isolation and first strand cDNA synthesis

Total RNA was isolated from roots, meristems, seedlings, leaves, flowers before fertilization (immature flowers), seeds 1–3, 3–5, 5–10, 10–15 days after fertilization (DAF), using TRI REAGENT 3 (SIGMA) according to the instructions of the manufacturer. First strand cDNA synthesis was performed using 1.0 μg total RNA, 0.5 μg 3′ RACE Adapter primer, 5′-5 GGCCACGCGTCGACTAGTAC (T)17-3′ (Invitrogen), 1 mM dNTPs and 200U of Superscript II (Invitrogen) in 20 μL total volume, according to the specifications of the manufacturer.

### Protein sequence analysis

Multiple alignment was created with ClustalW. The phylogenetic tree was calculated using MEGA 5.0 software [49] by the Neighbor-Joining Method with p-distance correction [50]. Bootstrap values were obtained from 1000 bootstrap replicates. The 3D-structures were predicted using swiss-model (http://swissmodel.expasy.org/) and visualized with FirstGlance in Jmol (http://oca.weizmann.ac.il/oca-docs/fgij/slides.htm). Accession numbers of sequences used for alignments and phylogenetic analysis are indicated in Additional file 1: Table S1.

### Genomic organization

The *DME* genomic sequences of *Brachypodium distachyon* (Bradi4g08870.1), *Oryza sativa* (01 g11900.1) and *Zea mays* (GRMZM2G123587) were downloaded from the Phytozome database (http://www.phytozome.net/). The sequence of *HvDME* was obtained from GenBank (BAC 273i4, accession number FM164415.1). Genomic organization of exons and introns was obtained using the mRNA-to-genomic alignment Spidey tool, in NCBI (http://www.ncbi.nlm.nih.gov/spidey). Detection of retroelements was performed with the MASiVE and LTRharvester tools (http://tools.bat.ina.certh.gr/masive/) (http://tools.bat.ina.certh.gr/ltrharvester/) and homology was visualized with Circoletto (http://tools.bat.ina.certh.gr/circoletto/). Statistically significant prediction for CpG islands was performed using the online predictor, which is part of the sequence manipulation suite at http://www.bioinformatics.org/SMS/index.html.

### Expression analysis of HvDME in tissues and under drought

Qualitative RT-PCR and quantitative real-time RT-PCR was performed with cDNA synthesized from 1 μg of total RNA from roots, stems, meristems, leaves, immature flowers, seeds 1–3 DAF, 3–5 DAF, 5–10 DAF, 10–15 DAF and aerial parts of seedlings after drought treatment. For real-time PCR, each sample reaction was set up in a PCR reaction mix (20 μl) containing 5 μl of the 1:50 diluted cDNA, 0.25 μM of each primer and 1× Platinum SYBR Green qPCR Supermix-UDG (Invitrogen, Paisley, UK) and using the Corbett Rotor Gene 6000. Each reaction was performed in triplicate. General thermocycler conditions were 50°C for 2 min, 95°C for 2 min, then 42 cycles [denaturing at 95°C for 20 sec, annealing at 56°C for 25 sec, extension at 72°C for 25 sec], then 72°C for 10 min. To identify the PCR products a melting curve was performed from 65°C to 95°C with observations every 0.2°C and a 10-s hold between observations. Relative quantification was performed using actin as the reference gene and *HvActinF/HvActinR* as primers. Primers used in expression analysis correspond to non-conserved regions and are shown in Additional file 2: Table S2.

### DNA methylation assays

Genomic DNA was prepared from control and drought-treated seedlings (Caresse and Demetra) with Qiagen columns following the protocol of the manufacturer (Qiagen Plant genomic DNA kit). Cytosine DNA methylation was analyzed by restricting 1 μg of genomic DNA from each sample with the methylation-dependent enzyme *Mcr*BC (NEB Biolabs), according to the manufacturer’s instructions, and PCR-amplifying equal quantities of *Mcr*BC-treated and untreated samples. Primers used are shown in Additional file 2: Table S2.

## Results

### HvDME protein sequence analysis

**Figure 1:**
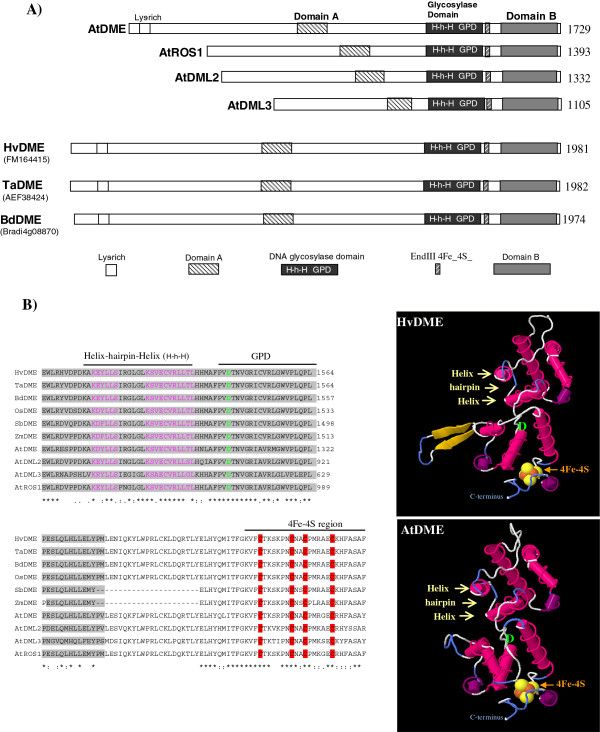
**Schematic view of DME-family DNA glycosylases and predicted tertiary structure of HvDME and AtDME. A)** Proteins, HvDME (FM164415), TaDME (AEF38424.1), BdDME (Bradi4g08870), AtDME (NP_196076.2), AtROS1 (AAP37178.1), AtDML2 (NP_187612.5) and AtDML3 (NP_195132.3) are depicted with white rectangles. White box, lysine-rich region; black box, glycosylase domain; hatched box, A domain; grey box, B domain; grey hatched box, 4Fe-4S binding domain; H-h-H, helix-hairpin-helix; GPD, glycine-proline rich region and conserved aspartate residue. **B)** Left: Amino acid sequence alignment of the glycosylase domain of DME-type proteins. Right: Predicted tertiary structure of HvDME and AtDME glycosylase domains. Helix-hairpin-helix is indicated with yellow arrows, the conserved aspartate (D) is shown in green and the 4Fe-4S is shown in orange-yellow.

A gene sequence with accession number FM164415.1 corresponding to a barley *HvDME* gene from the cultivar Morex was retrieved from GenBank. This gene sequence had been deposited, annotated and described in GenBank (Langen, G., Pang, J., Brueggeman, R. and von Wettstein, D., May 2008). The sequence is contained in the BAC clone BAC 273i4, and encompasses 17 exons and 16 introns in a total size of 26642 bp. BAC 273i4 also contains 6300 bp 5′upstream from the ATG translational start and 6735 bp 3′downstream from the TAG translational stop codon. *HvDME* harbors 5946 bp of coding sequence which translates to a putative protein of 1981 aa. The DME-family amino acid sequences of Arabidopsis and other cereals were retrieved from the GenBank and Phytozome (Additional file 1: Table S1) and an alignment was constructed (Additional file 3). HvDME has a common structure with the other DME-type DNA glycosylases in that it harbours a lysine-rich region at the N-terminus, ensued by Domain A which is followed by the DNA glycosylase domain [including the helix-hairpin-helix motif (HhH) and the glycine-proline rich region flanked by a conserved aspartate (GPD)], the EndIII _4Fe-4Se domain and Domain B (Figure 1A and Additional file 3). HvDME has a high degree of homology with TaDME2 (AEF38424.1) (96.4% identity), BdDME (Bradi4g08870.1) (77.5% identity), OsDME (01 g11900.1) (70% identity), and less with ZmDME1 (GRMZM2G123587) (52% identity) and SbDME1 (08 g008620.1) (49% identity) (Additional file 3). Near absolute conservation is observed among cereals in the region spanning the DNA glycosylation domains and the the EndIII _4Fe-4Se domain. About 65% similarity exists between the DNA glycosylation domain of HvDME and the DNA glycosylation domain of AtDME (Additional file 3). The tertiary structure of the glycosylase domain of HvDME and AtDME, respectively, was predicted using Swissmodel (Figure 1B) and found to be very much alike except for a beta-strand loop in the HvDME, not predicted for the AtDME protein. The spatial orientation of the helix-hairpin-helix, the conserved aspartate (D), and the adjacent four-cysteine region binding a putative 4Fe-4S cluster were nearly identical (Figure 1B).

**Figure 2:**
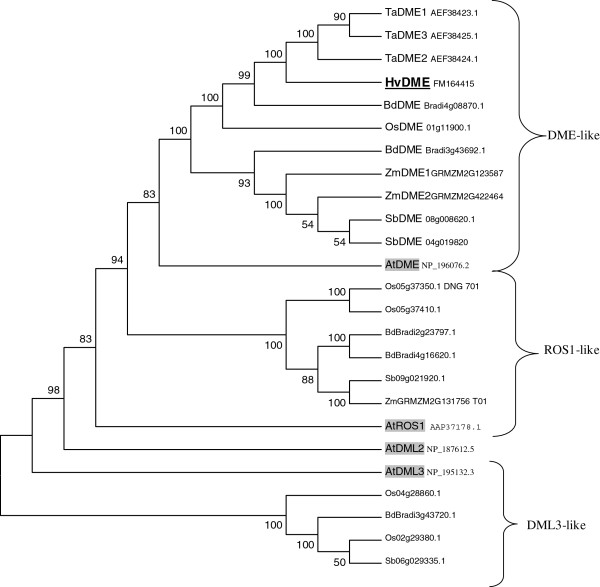
**Phylogenetic analysis of DME-family DNA glycosylases from monocots and dicots.** Phylogenetic tree showing the classification of DME-type DNA glycosylases. The sequences used and their accession numbers are shown in Additional file 1: Table S1. Barley HvDME is in bold and Arabidopsis AtDME, AtROS1, AtDML2 and AtDML3 are grey-shaded. Numbers indicate bootstrap values (1000 = 100%).

A phylogenetic tree constructed using all known DME-family sequences from barley, wheat, brachypodium, rice, maize, sorghum and Arabidopsis showed that HvDME belongs in a cluster with the DME homologues from other cereals, all being more closely related to the AtDME, whereas is more distantly related to other DNA glycosylase members which group together with ROS1, DML2 and DML3 homologues (Figure 2). Barley homologues of the ROS1, DML2 and DML3 proteins have not been identified thus far.

### Comparative genomic analysis of *cereal DME* genes

**Figure 3:**
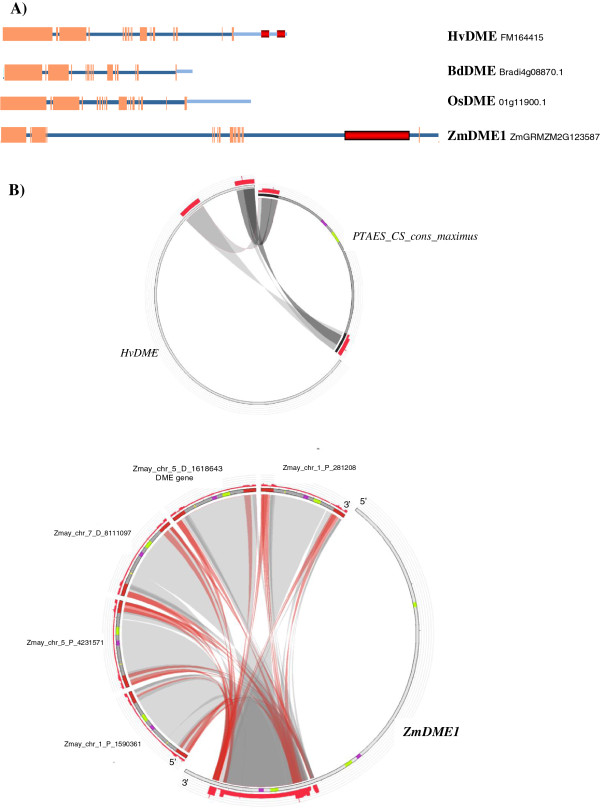
**Genomic analysis of cereal *DME *genes. A)** Schematic view of cereal *HvDME* (FM164415), *BdDME* (Bradi4g08870), *OsDME* (01 g11900.1) and *ZmDME* (GRMZM2G123587) genes. Exons are depicted with orange boxes and introns with blue lines. 3’ untranslated regions are shown as light blue lines. Regions within the 3’ untranslated region of *HvDME* and within the intron of *ZmDME* where retrotransposon sequences were found are highlighted in red. **B)** Sequence similarity of *HvDME* and *ZmDME* with *Copia* retrotransposons, visualized in Circoletto (http://tools.bat.ina.certh.gr/circoletto/) after blast analysis with MASiVE (http://tools.bat.ina.certh.gr/masive/) and LTRphyler (http://tools.bat.ina.certh.gr/ltrphyler/). Upper circle: Full length *HvDME* gene (left); the *Copia PTAES_CS_cons_maximus* Sirevirus retrotransposon (right). The 5’ and 3’ LTR regions of the retrotransposon are shown with bold black lines. The regions of retrotransposon homology between the LTRs and the 3’ downstream region of *HvDME* are marked in red. Lower circle: Full length *ZmDME1* gene (right); the maize *Copia Ji* Sirevirus retrotransopson element in different chromosomal locations of the maize genome (left). The *ZmDME* gene resides at the Zmay_chr_5_D_1618643 site (chromosome 5, sense strand, position 1618643 bp from the chromosomal start). Regions of homology are shown in red. Coding regions of retrotransposon integrase and reverse transcriptase are in purple and yellow-green, respectively. Dark-grey and light- grey interconnecting ribbons depict regions of high and low similarity, respectively.

The *HvDME* gene sequence contained in the BAC clone BAC 273i4 has a total size of 26642 bp and harbors 17 exons and 16 introns (Figure 3A). *HvDME* also contains 6300 nt 5′upstream from the ATG translational start and 6735 nt 3′downstream from the TAG translational stop codon (Additional file 4 and Additional file 5). The gene sequences of Brachypodium *BdDME* (Bradi4g08870.1) and rice *OsDME* (01 g11900.1) also contain 17 exons and 16 introns, respectively. The exons are of similar sizes whereas some variation is observed among introns regarding relative size and position (Figure 3A and Additional file 4). *ZmDME* (GRMZM2G12358) consists of 16 exons and 15 introns with two large introns, 4, and 14. Using the MASiVE bioinformatics tool for detection of retroelements (http://tools.bat.ina.certh.gr/masive/) [51], two sites in the 3′downstream untranslated region of the *HvDME* gene were found to have a high degree of similarity with the *PTAES_CS_cons_maximus Copia* Sirevirus retroelement. Specifically, the 5′LTR region (1437 nt) and the 3′LTR region (1161 nt) of the *PTAES_CS_cons_maximus* element were detected at 1696 nt and 5540 nt downstream from the translational stop of *HvDME*, within 21600–23037 nt and 25444–26605 nt, respectively (Figure 3B and Additional file 5). Interestingly, a full length (9591 nt) maize Sirevirus retrotransposon, *Copia Ji,* was found to have high similarity with a fragment of intron 14 (from 34003 to 43593 nt) of the maize *ZmDME1* (GRMZM2G123587) gene. Highest homology was with the 5′LTR and 3′LTR regions of the retrotransposon contained in 42135–43593 nt and 34003–35337 nt of the *ZmDME1* gene (Figure 3A, 3B and Additional file 4). The *Copia J*i retrotransposon was detected in four more sites of the maize genome as indicated in Figure 3B which upon further inspection in Ensemble (http://plants.ensembl.org/index.html) were found to be intergenic regions.

### miRNA target analysis for *DME* from barley and other cereals

The report that an Arabidopsis DME-family DNA glycosylase homologue, *AtDML3,* is regulated by the miRNA miR402 [52], prompted us to perform small RNA target analysis, *in silico*, for detecting putative small RNA targets on the *HvDME* gene sequences. Similar analysis was performed with the *DME* gene homologues from Brachypodium and rice using the psRNATarget (Plant small RNA Target) tool (http://plantgrn.noble.org/psRNATarget).

**Figure 4:**
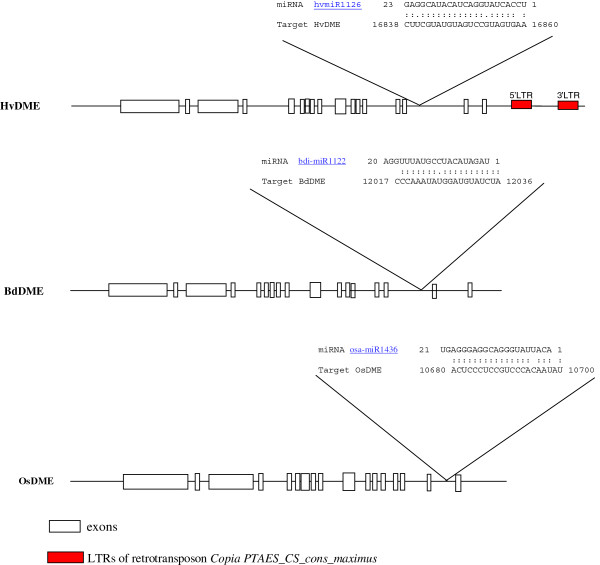
***In silico *miRNAs analysis.** Putative miRNA target sites in *HvDME* (FM164415), *BdDME* (Bradi4g08870), and *OsDME* (01 g11900.1). MiRNA and target complementarities are depicted schematically above the sequences. White boxes represent exons; black lines represent introns and upstream and downstream regions. Identitical nucleotide matching in the miRNA-target hybrid is depicted by double dot (:). Permitted G-U matches are depicted by single dot (.).

Inspection of the coding region of the *DME* genes did not identify target sites with sufficient complementarity to plant miRNAs. However, upon examining genomic sequences, near 100% complementarity was detected between miRNA *HvmiR1126* and intron 15 (16644–19175 nt) of the *HvDME* gene sequence (Figure 4). 22 nt out of 23 nt of miRNA *HvmiR1126* matched with the region 16838 nt-16860 nt of intron 15. Likewise near 100% complementarity was detected between the Brachypodium miRNA, *BdmiR1122*, and intron 15 of the *BdDME* gene and between the rice miRNA *OsmiR1436* and intron 16 of the *OsDME* gene (Figure 4).

### Expression analysis of *HvDME* in different tissues and seed developmental stages

**Figure 5:**
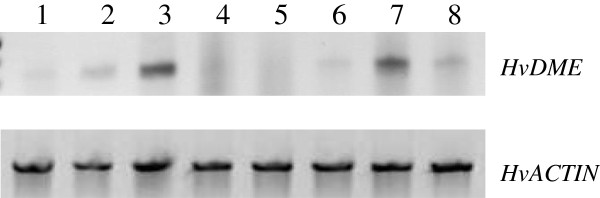
**Qualitative RT-PCR expression analysis of *HvDME *in vegetative and reproductive tissues in the cultivar Carina.** 1, roots; 2, meristems; 3, 7 day-old seedlings; 4, leaves; 5, stamens; 6, immature flowers (before fertilization); 7, seeds 1–3 days after fertilization; 8, seeds 3–5 days after fertilization. *HvActin* was used as the positive control.

Expression analysis of *HvDME* was examined in vegetative and reproductive tissues from the barley cultivar Kos (a medium seed-size cultivar) by qualitative RT-PCR (Figure 5). *HvDME* transcript is detectable in all tissues examined except from mature leaves and stamens. Highest expression of *HvDME* was in seedlings and in seeds 1–3 (DAF) which declined in seeds at 3–5 DAF.

**Figure 6:**
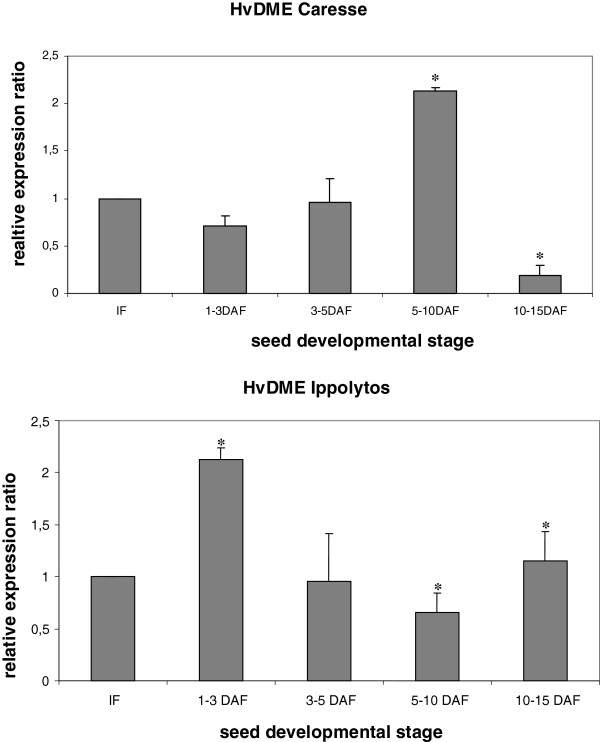
**Quantitative real time RT-PCR analysis of *HvDME *during seed development in Caresse and Ippolytos.** Expression values were normalized to those of *HvActin*. The relative expression ratio of each sample is compared to the control group which was immature flowers. IF, Immature flowers; 1–3, 3–5, 5–10, 10–15 DAF (seeds at 1–3, 3–5, 5–10, 10–15 days after fertilization). Data represent mean values from two independent experiments with standard deviations. Values significantly different (P < 0.05) from the control group (IF) are marked with an asterisk.

Expression analysis of *HvDME* in different seed developmental stages in two different cultivars with different seed size, Caresse (large-seed) and Ippolytos (small-seed), was performed by quantitative real time RT-PCR. In the cultivar Caresse, *HvDME* expression is higher by about 2 fold in the 5–10 DAF stage whereas it is lower by about 4 fold in the 10–15 DAF stage, as compared to the unfertilized immature flower (IF) (Figure 6). In the cultivar Ippolytos *HvDME* expression increased by about 2 fold in the 1–3 DAF seed stage as compared to the immature flower (IF) whereas in the later seed stages *HvDME* expression did not seem to change (Figure 6).

### Expression analysis of *HvDME* in response to drought stress

**Figure 7:**
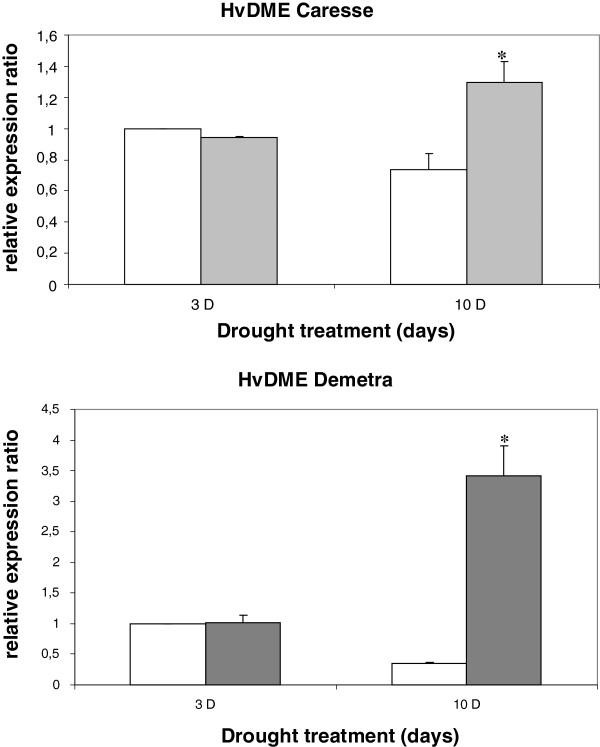
**Expression analysis of *HvDME *under drought.** Quantitative real time PCR analysis of *HvDME* at 3 and 10 days after drought treatment of Caresse(drought-sensitive) and Demetra (drought-tolerant) 7-d-old seedlings. White bars, untreated plants; grey bars and light grey bars, drought-treated plants. Expression values were normalized to those of *HvActin.* Relative expression ratio of each sample was compared to the control group which was untreated plants, 3 days, and was assigned the value of 1. Data represent mean values from two independent experiments with standard deviations. Values significantly different (P < 0.05) from the untreated plants are marked with an asterisk.

Environmental stress such as drought can have important consequences in proper plant development and seed yield. The drought response is a complex process involving the action of different structural genes and gene transcription factors [53,54]. In addition epigenetic factors such as histone deacetylases, histone methyltransferases and demethylases, and miRNAs have been implicated in the response [44,47,55-58]. Therefore, another epigenetic regulator such as the *DME* gene may also be required for regulating the gene expression programmes participating in drought response. Expression of *HvDME* was investigated upon conditions of drought, in two barley cultivars with different tolerance to drought. Real-time PCR was employed to examine *HvDME* transcript accumulation in 7-d-old seedlings at 3 and 10 days after drought treatment in the drought-sensitive cultivar, Caresse, and the drought-tolerant cultivar, Demetra. A sound induction of ~ 10 fold was observed for *HvDME,* after 10 days of drought in the drought-tolerant cultivar Demetra as compared to the untreated control plants after 10 days (Figure 7). *HvDME* transcript levels increased by ~ 2 fold after 10 days of drought in the drought-sensitive cultivar Caresse (Figure 7).

### DNA methylation analysis of the *HvDME* gene

In order to examine the DNA methylation pattern of the *HvDME* gene and uncover potential links to gene expression differences between drought-treated and untreated plants, the DNA methylation profile of *HvDME* in control and drought conditions was analysed using an *Mcr*BC-PCR assay. *Mcr*BC is a methylation-sensitive restriction enzyme that cleaves DNA containing methylcytosine on one or both strands, recognizing two half-sites of the form (G/A)mC. After *Mc*rBC treatment, methylated DNA will be digested and will not be amplified by PCR, while unmethylated DNA will not be cleaved and will result in PCR product.

**Figure 8:**
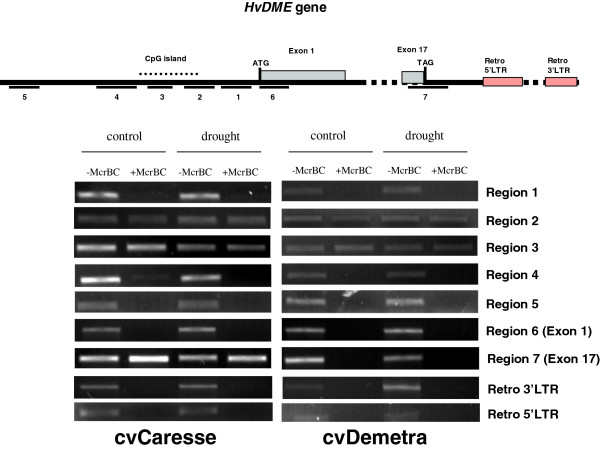
**DNA methylation of *HvDME.*** DNA methylation assays in 10 day drought-treated and untreated Caresse and Demetra seedlings. Upper: A schematic view of the *HvDME* gene depicted as a bold black line. Pale blue boxes, exons; pink boxes, LTRs. The regions used for *Mcr*BC-PCR are underlined and numbered. ATG and TAG codons are indicated. Lower: Analysis of PCR amplification of *Mcr*BC-digested and undigested genomic DNA on agarose gels. Genomic DNA was digested with *Mcr*BC and PCR amplification followed. (−), no *Mcr*BC; (+), digestion with *Mcr*BC.

We analyzed Caresse and Demetra seedlings 10 days after drought treatment since this tissue showed the highest difference in expression (~2 fold and 10 fold increase, respectively) compared to untreated tissue (Figure 8). Four regions in the promoter of *HvDME* (regions 1, 2, 3 and 4), two coding regions (regions 6 and 7) and a region located 3780 nt upstream from the translation start site (region 5) were examined. No obvious bands regarding region 1 (−76 to −432 from the ATG start) and region 4 (−1381 to −1781) were detected, indicating that these regions are cytosine methylated in both cultivars. On the other hand, the presence of PCR bands for region 2 (−501 to −854) and 3 (−883 to −1167), both being part of a CpG island, suggests that they are unmethylated. Region 6 (+9 to +755 from the ATG start) containing the 5′end of exon 1 (747 nt), is methylated in both cultivars as indicated by the absence of PCR products. Interestingly, region 7 (containing part of exon 17 with 125 nt, and part of the 3′ untranslated region with 292 nt) is only methylated in Demetra, whereas a strong PCR band indicates that it is unmethylated in Caresse. Region 5 which is predicted to be a CpG island is heavily methylated in both Demetra and Caresse. Taken together, these data demonstrate the presence of cytosine methylation in both the promoter and gene-body regions of the *HvDME* gene in the two cultivars.

Using the McrBc assay no differences in the DNA methylation status between control and drought-treated plants were detected in the regions examined in either cultivar suggesting that there may not be an association between DNA methylation and increased *HvDME* expression under drought conditions.

Two *HvDME* 3′ downstream regions (at 1696 nt and 5540 nt from the translational stop) found to contain the 5′ and 3′ LTR regions of a *Copia* retrotransposon, *PTAES_CS_cons_maximus,* were also examined for the presence of DNA methylation. The 5′ and 3′ LTRs were shown to be methylated in control and drought-treated plants in both cultivars (Figure 8).

## Discussion

In the current study we present the sequence analysis, phylogenetic analysis, expression profiles, genomic organization, promoter analysis and DNA methylation patterns of a barley gene encoding a putative HvDME protein.

### Sequence analysis

Comparison of amino acid sequences from different DME-family proteins from the dicot Arabidopsis and different monocot species revealed that the HvDME protein has a common modular structure as the other members of the DNA glycosylase superfamily. Furthermore, it contains the two unknown domains A and B, found only in DME-family DNA glycosylases. Phylogenetic analysis of DME-family sequences from different monocots revealed three major cereal clades representing homologues of the AtDME, AtROS1, and AtDML3 proteins. HvDME shows highest sequence similarity with the grass sequences, TaDME1 (AEF38423.1), TaDME2 (AEF38424.1) TaDME3 (AEF38425.1), BdDME (Bradi4g08870.1), and OsDME (01 g11900.1) which share over 70% identity and group together in a subcluster that is closer related to the Arabidopsis DME.

Analysis of the *HvDME* genomic organization*,* revealed a high degree of similarity with its grass homologues, *BdDME* (Bradi4g08870.1) and *OsDME* (01 g11900.1). The presence of sequences from an LTR *Copia* Sirevirus retroelement in the 3′ untranslated region of barley *DME* is consistent with the recent finding that 86% of the barley genome is composed of mobile elements or other repeat sequences the majority of which consists of LTR retrotransposons [59].

In Arabidopsis, the AtDML3 homologue has been suggested to be regulated by the stress-induced miRNA 402 during seed germination under stress conditions [52]. MiRNAs and small RNAs have been widely studied in plants in the past several years and have been shown to play important roles in various aspects of plant development [60]. Although initial studies focused on Arabidopsis, intense investigations were soon extended to agronomically important crops such as cereal monocots [57,58,61-64]. A search for miRNA targets on the *HvDME* sequence identified miRNA *HvmiR1126* in intron 15 of the *HvDME* gene sequence at near 100% complementarily. Curiously, similar complementarity was detected between the Brachypodium miRNA, *BdmiR1122*, and intron 15 of the *BdDME* gene and between the rice miRNA, *OsmiR1436,* and the last intron of the *OsDME* gene, respectively. The high complementarity of these miRNAs and their targets might imply that they are functionally significant. Intronic miRNAs have been the object of recent studies and demonstrated to play important roles in the regulation of genes in mammals [65]. In plants, studies on intronic miRNAs have just started to emerge [66-68]. Certainly further investigations will be needed to unveil the possible significance of *HvmiR1126*.

### *Expression* analysis

Our data on *HvDME* expression in barley vegetative, reproductive and seed tissues agrees with data retrieved from PLEXdb (http://www.plexdb.org/) concerning the expression of the *DME* gene from different barley cultivars such as Morex and Golden Promise showing *DME* mRNA presence throughout plant development. Moreover it concurrent with recent datasets from the large-scale genome and transcriptome analysis of barley [59] showing the *HvDME* transcript (MLOC_17707.1) present in Morex seedlings, developing tillers, immature flowers and 4 DAF embryos, and in seeds of 5 and 15 DAF. Ippolytos resembles Morex which is another small-seed cultivar (31.5 gr per thousand grains) as gene expression in 15 DAF seeds is about 1.5 higher than in 5 DAP seeds (ftp://ftpmips.helmholtz-muenchen.de/plants/barley/public_data/).

In barley, endosperm cellularization begins at approximately 4 DAF and ends at 6–8 DAF, when the seed maturation process begins [69,70]. It might be possible that the differences in *HvDME* mRNA accumulation between Caresse (large-seed) and Ippolytos (small-seed) during these critical stages of endosperm development affect gene expression programmes associated with the processes of cellularization and seed filling and ultimately with the size of seed. At 10–15 DAF the barley seed has entered the maturation process with high starch synthesis rates and protein accumulation characterizing this seed storage stage. The differential expression of *HvDME* at the 10–15 DAF seed stage between the two cultivars may reflect varied requirements for metabolic enzyme-encoding genes playing roles in starch and protein synthesis and storage. Certainly the exact role of *HvDME* in the two cultivars will be elucidated by future functional analysis of the *HvDME* gene.

*HvDME* expression was examined under conditions of drought and was found to be substantially induced in drought-treated seedlings especially in the drought-tolerant cultivar. Interestingly, in the drought-tolerant cultivar, Demetra, there is a marked increase of *HvDME* expression of ~ 10 fold, after 10 days of drought, whereas only ~ 2 fold increase was observed for the drought-sensitive cultivar Caresse. This suggests a possible role for the *HvDME* gene in the drought response in a cultivar-dependent manner. Although studies on *DME* glycosylases under stress are currently scarce, a recent study of response to metal stress in rice revealed induction of the rice *DME* gene in soils with elevated mercury, cadmium and copper [71]. Perhaps induction of the *DME* gene is required in cereals in order to cope with the stress imposed on the plant by unfavourable soil composition and low water content.

### DNA methylation

Three regions of the *HvDME* promoter (region 1, region 4, and region 5) were found to be methylated in both cultivars under control and drought conditions. On the other hand, two regions that are part of the CpG island (region 2 and region 3) of the *HvDME* promoter were unmethylated in all instances. It seems that the CpG island is in an unmethylated state while the neighbouring regions to the CpG island are methylated, which is in accordance with previous reports demonstrating that CpG islands are typically in a nonmethylated state in an otherwise heavily methylated genome [72]. The finding that an active gene is promoter-DNA methylated seems inconsistent with studies in Arabidopsis and rice that have associated promoter DNA methylation with gene repression [5,73]. On the other hand, genome-wide mapping of DNA cytosine methylation in rice revealed that 8.1% of active genes were methylated within their promoter [74]. Similarly, the cold-induced gene *ZmDREB1* in maize retains some cytosine methylation marks within the promoter region after cold induction [75]. In soybean, a region of the promoter of a salinity induced gene was demethylated upon salinity stress, whereas a neighbouring region remained hypermethylated supporting the suggestion that cytosine methylation is region specific [76]. In addition, it was reported that while cytosine methylation is a repressive mark, H3K4me2 alters the chromatin structure to a form permissive for initiation of transcription even in the presence of cytosine methylation [73]. In barley too, other factors such as histone modifications in interplay with promoter cytosine methylation may be critical in governing *HvDME* expression.

Gene-body (transcribed region) DNA methylation was also investigated for a fragment covering part of exon 1 and a fragment including exon 17 and a part of the 3′UTR. Exon 1 was shown to be methylated in both Demetra and Caresse, whereas exon 17 was methylated only in Demetra. DNA methylation in *HvDME* gene-body is consistent with a number of reports demonstrating the presence of gene-body methylation in plants. DNA methylation within transcribed regions has been detected in about a third of Arabidopsis [77,78] and rice [73,79] genes. Substantial expression of *HvDME* in various tissues and its enrichment for gene-body DNA methylation is in accordance to previous studies in Arabidopsis showing that highly and moderately expressed genes are more likely to be DNA methylated within the gene-body region [77,80]. Additionally, it is in agreement with a recent study where the single-base methylome of wild- and cultivated- rice revealed that promoter DNA methylation is associated with gene repression whereas gene-body DNA methylation is associated with gene expression [79].

Apart from its importance in seed development and stress copying mechanisms, understanding the regulation of *DME* genes could have important implications in nutrition and health. In a very recent investigation attempting to analyze the effects of wheat and barley *DME* genes on the levels of immunoactive prolamins associated with gluten-intolerance and celiac disease, wheat *DME* was RNAi-downregulated resulting in decreased expression of endosperm prolamins [39]. These efforts could lead to improved cereal cultivars producing safe cereal products for gluten-sensitive individuals [34-39].

During the past several years our group has studied different epigenetic chromatin regulators implicated in gene activation or gene repression in barley [40-48]. Ongoing efforts to further our knowledge on epigenetic regulatory mechanisms impacting seed development, nutritional seed content, and the plants′ resistance to abiotic stresses such as drought, could lead to breeding for improved Triticeae varieties.

## Conclusions

A *DME* homologue was characterized in barley and the encoded protein was found to group together with other cereal DME-family proteins more closely related to AtDME and more distantly related to AtROS1, AtDML2 and AtDML3. *HvDME* contains remnants of a *Copia* retroelement in its 3′downstream region which maybe important for its regulation. *HvDME* displayed differential expression during seed development in two cultivars varying in seed size, implying a role in endosperm development and seed maturation. Moreover, *HvDME* expression is markedly increased in dehydrated seedlings in a drought-tolerant cultivar pointing to a role in response to abiotic stress such as drought. Finally, differential DNA methylation in different regions of the gene-body in two different cultivars suggests epigenetic regulation of the *HvDME* gene. The study of a barley *DME* gene will contribute to our understanding of epigenetic regulation during seed development and in response to abiotic stresses in cereal crops of high agronomic value.

## Competing interests

The authors declare that they have no competing interests.

## Authors’ contributions

AK conceived and designed the experiments, performed RNA isolation, qualitative and quantitative real time PCR assays, protein sequence analysis, phylogenetic analysis, genomic organization analysis and wrote the manuscript. VD performed promoter analysis, designed and performed DNA methylation experiments and participated in the writing of the manuscript. AA participitated in the DNA methylation studies. AST revised the manuscript and directed the whole study. All authors read and approved the final manuscript.

## Supplementary Material

Additional file 1: Table S1 Accession numbers of HhH-GPD DNA glycosylase superfamily members. Click here for file

Additional file 2: Table S2 Primers used in expression and DNA methylation analyses. Click here for file

Additional file 3 **Amino acid sequence alignment of DME protein sequences from *Hordeum vulgare*, HvDME (FM164415.1); *Triticum aestivum*, Ta DME2 (AEF38424.1); *Brachypodium distachyon*, BdDME (Bradi4g08870.1); *Oryza sativa*, OsDME (01 g11900.1); *Zea Mays*, ZmDME (GRMZM2G123587); *Sorghum bicolor*, SbDME (08 g008620.1); *Arabidopsis thaliana*, AtDME, AtROS1, AtDML2, AtDML3.** Identical amino acids are shown with asterisks and similar amino acids with dots. The DNA glycosylase domain is indicated in grey highlights, the helix-hairpin-helix region is overlined and marked in purple, the GPD region is overlined and the conserved aspartic acid residue is marked in green. The four cysteine residues forming the 4Fe-4S cluster are shown in red. The conserved lysine-rich region, and the A and B regions are also overlined. Click here for file

Additional file 4 **Genomic organization of cereal *DME* genes.** Click here for file

Additional file 5 **The sequence of clone BAC 273i4, containing the *HvDME* gene.** ATG and TAG translational start and stop codons, respectively, are shaded in pink. 5’ and 3’ LTR regions of the *Copia* sirevirus retrotransposon *PTAES_CS_cons_maximus* are shaded in grey. Click here for file
