# Supplementary material for: The study of a barley epigenetic regulator, HvDME, in seed development and under drought
Source: BMC Plant Biol. 2013 Oct 31;13:172. doi: 10.1186/1471-2229-13-172 (PMC4228467; doi:10.1186/1471-2229-13-172)
Supplement: Additional file 1: Table S1 — Accession numbers of HhH-GPD DNA glycosylase superfamily members. [file 1471-2229-13-172-S1.doc]

**Additional Files**

**Additional File 1**

Table 1. Accession numbers of HhH-GPD DNA glycosylase superfamily members

| **ORGANISM** | **ACCESSION No and NAME** | **Family** |
| --- | --- | --- |
|  |  | **EndoIII-family** |
| Escherichia coli | YP_491160 AdenineDNA glycosylase (MutY) |  |
| Escherichia coli | NP_288069.1 EndoIII DNA glycosylse |  |
| Homo sapiens | O15527 8-oxoguanine DNA glycosylase |  |
|  |  |  |
|  |  | **DME-family** |
| Arabidopsis thaliana | NP_196076.2 AtDME | **DME** |
| Hordeum vulgare | FM164415.1 HvDME |  |
| Triticum aestivum | AEF38423.1 TaDME1 |  |
| Triticum aestivum | AEF38424.1 TaDME2 |  |
| Triticum aestivum | AEF38425.1 TaDME3 |  |
| Brachypodium distachyon | Bradi4g08870.1 BdDME |  |
| Oryza sativa | 01g11900.1 OsDME |  |
| Zea mays | ZmGRMZM2G123587 ZmDME1 |  |
| Zea mays | ZmGRMZM2G422464 ZmDME2 |  |
| Sorghum bicolor | 08g008620.1 SbDME1 |  |
| Sorghum bicolor | 04g019820.1 SbDME2 |  |
| Arabidopsis thaliana | AAP37178.1 AtROS1 | **ROS1** |
| Oryza sativa | Os05g37350.1, DNG 701 |  |
| Oryza sativa | Os05g37410.1 |  |
| Brachypodium distachyon | BdBradi2g23797.1 |  |
| Brachypodium distachyon | BdBradi4g16620.1 |  |
| Sorghum bicolor | Sb09g021920.1 |  |
| Zea mays | ZmGRMZM2G131756 |  |
| Arabidopsis thaliana | NP_195132.3 AtDML3 | **DML3** |
| Oryza sativa | Os04g28860.1 |  |
| Oryza sativa | Os02g29380.1 |  |
| Brachypodium distachyon | BdBradi3g43720.1 |  |
| Sorghum bicolor | Sb06g029335.1 |  |
| Arabidopsis thaliana | NP_187612.5 AtDML2 | **DML2** |
