# Supplementary material for: The study of a barley epigenetic regulator, HvDME, in seed development and under drought
Source: BMC Plant Biol. 2013 Oct 31;13:172. doi: 10.1186/1471-2229-13-172 (PMC4228467; doi:10.1186/1471-2229-13-172)
Supplement: Additional file 2: Table S2 — Primers used in expression and DNA methylation analyses. [file 1471-2229-13-172-S2.doc]

**Additional File 2**

Table 2. Primers used in expression and DNA methylation analyses

| **Primer name** | **5’ - 3’ Primer sequence** | **Size of fragment (bp)** |
| --- | --- | --- |
| **Expression analysis** |  |  |
| HvDME F | AGATGATGCATAGCCCGTCAAG | 230 |
| HvDME R | GAGCATAAATCTGTGTAGTAATG |  |
| HvActin F | CTGACGGTGAGGACATCCAG | 235 |
| HvActin R | CGTGAGGATACCTCTCTTGGA |  |
|  |  |  |
| **DNA Methylation analysis** |  |  |
| HvDMEm1F | TCGCAGGAAAGGATAAATGC | 356 |
| HvDMEm1R | ACTCGTGAGGAAAAGCAGGA |  |
| HvDMEm2F | TAATCAAGTGGAGGGCCTGT | 353 |
| HvDMEm2R | CCGTGGATGGCTAAATCACT |  |
| HvDMEm3F | CCCCTCACTCGATCCTACTC | 284 |
| HvDMEm3R | GGGAACGCTCAAAGTCAGAT |  |
| HvDMEm4F | GAACCATTTCACCGTTGGTC | 400 |
| HvDMEm4R | AACCTCGCATGTGCTTCTTC |  |
| HvDMEm5F | ATAGCTTGCGCCACTTTTGT | 327 |
| HvDMEm5R | GTGTCGTCCTCCCAGGTCTA |  |
| HvDMEm6F | TTTTGGACAATGGCTTCCTC | 747 |
| HvDMEm6R | GCAGGCAAAAACTGTGCATT |  |
| HvDMEm7F | GTGTGAGGGGCTTTGATAGG | 417 |
| HvDMEm7R | ACAAACAGAATGGCCCAGAC |  |
| 5’LTR F | GTAGAATGCAACATGCAAGAACAAG | 1780 |
| 5’LTR R | GTTGACGTTGACTTTGATATGAAG |  |
| 3’LTR F | CTAAGCTCGCGACCACCATC | 1298 |
| 3’LTR R | GAATTCGGTGTGTCCTGAAG |  |
