# Supplementary material for: The study of a barley epigenetic regulator, HvDME, in seed development and under drought
Source: BMC Plant Biol. 2013 Oct 31;13:172. doi: 10.1186/1471-2229-13-172 (PMC4228467; doi:10.1186/1471-2229-13-172)
Supplement: Additional file 3 — Amino acid sequence alignment of DME protein sequences from Hordeum vulgare, HvDME (FM164415.1); Triticum aestivum, Ta DME2 (AEF38424.1); Brachypodium distachyon, BdDME (Bradi4g08870.1); Oryza sativa, OsDME (01 g11900.1); Zea Mays, ZmDME (GRMZM2G123587); Sorghum bicolor, SbDME (08 g008620.1); Arabidopsis thaliana, AtDME, AtROS1, AtDML2, AtDML3. Identical amino acids are shown with asterisks and similar amino acids with dots. The DNA glycosylase domain is indicated in grey highlights, the helix-hairpin-helix region is overlined and marked in purple, the GPD region is overlined and the conserved aspartic acid residue is marked in green. The four cysteine residues forming the 4Fe-4S cluster are shown in red. The conserved lysine-rich region, and the A and B regions are also overlined. [file 1471-2229-13-172-S3.doc]

**Additional file 3**

Amino acid sequence alignment of DME protein sequences from *Hordeum vulgare*, HvDME (FM164415.1); *Triticum aestivum*, Ta DME2 (AEF38424.1); *Brachypodium distachyon*, BdDME (Bradi4g08870.1); *Oryza sativa*, OsDME (01g11900.1); *Zea Mays*, ZmDME (GRMZM2G123587); *Sorghum bicolor*, SbDME (08g008620.1); *Arabidopsis thaliana*, AtDME, AtROS1, AtDML2, AtDML3. Identical amino acids are shown with asterisks and similar amino acids with dots. The DNA glycosylase domain is indicated in grey highlights, the helix-hairpin-helix region is overlined and marked in purple, the GPD region is overlined and the conserved aspartic acid residue is marked in green. The four cysteine residues forming the 4Fe-4S cluster are shown in red. The conserved lysine-rich region, and the A and B regions are also overlined and highlighted in yellow, green and blue, respectively.

HvDME -MQGFGQWLPQSQSAADLYFSSI-MSSQLDTSIEMQTRNTE-VAVLENESAHSFGITNAA 57

TaDME -MQGFGQWLPQSQSAADLYFSSI-MSSQLDTSIEMQTRNTA-VAVLEDESAHSFGVTSAA 57

BdDME -MQDFGQWLPQSQNTADLYFSSM-VSSQLDTSIETQVRNSA-VALLEKESTHSFDIRDGA 57

OsDME -MQDFGQWLPQSQTTADLYFSSIPIPSQFDTSIETQTRTSA-VVSSEKESANSFVPHNGT 58

SbDME MQEGLGQRMHVP-HGINLCFATSSVPFQVESSIELGTGEVNPPVTSEKLPANSQAVN-DA 58

ZmDME MQEELGQRMHVP-HGINLCFSTSSELFQVESSIELGTAEVN-PVPSEKLSANSQAVRDDA 58

AtDME ----------------------------------------MQSIMDSSAVNATEATEQND 20

AtDML2 ---------------------------------------------MEVEGEVREKEARVK 15

AtDML3 -----------------------------------------------MLTDGSQHTYQNG 13

AtROS1 ---------------------------------------------MEKQ-RREESSFQQP 14

**Lysine-rich domain**

HvDME GPIEVTSNDAGTIIDNENVAEPTGGIDLNKTPPPKAKRKKHRPKVLK---PSKPPKPATP 114

TaDME GPIEVTRNDAGTIIDNENVAEPTGGIDLNKTPPPKAKRKKHRPKVLK---PSKPPKSATP 114

BdDME GHIEVIRNDAGAIKETENGAGLTDGIDLNKTPPPKTKRKKHRPKVLK---PSKTPKSATP 114

OsDME GLVERISNDAGLTEVVGSSAGPTECIDLNKTPARKPKKKKHRPKVLKDDKPSKTPKSATP 118

SbDME GALEG--------------------TDMNGKSVQKPKRKKHRPKVIKEGQSAKLQKPKTP 98

ZmDME GAVEG--------------------IDMNGKPVQKPKRKKHRPKVIKEGQSAKLQKPKTP 98

AtDME GSRQDVLEFDLNKTPQQKPSKRKRKFMPKVVVEGKPKRKPRKPAELPKVVVEGKPKRKPR 80

AtDML2 GRQP-----------------------------------------------ETEVLHGLP 28

AtDML3 ETK--------------------------------------------------NSKEHER 23

AtROS1 PWIP-----------------------------------------------QTPMKPFSP 27

HvDME KPSKAKEEKPSGKRKYVRKN--TPAGQPPPEQIAGSHCRANLKPAKRSLNFEGEVPQENT 172

TaDME KPSKAKEEKPSGKRKYVRKN--MPAGQPPSEQTAESHRKATVKPAKRSLNFEGEVPQENT 172

BdDME KPSKEKEERPSGKRKYVRKN--TPAGQPPPEQAANSHCSAKLKPAMRCLNFNGAVPQENP 172

OsDME IPSTEKVEKPSGKRKYVRKK--TFPGQPPAEQAASSHCRSELKSVKRSLDFGGEVLQEST 176

SbDME KPPKENGNQPTAKRKYVRRKGLSAPAEQIPSGGADTQTTAKPGVAQRCLDFDVEDQHGHL 158

ZmDME KPPKENGNQPTGKRKYVRKKGLSTPAKQIPSEGADTHTRAKPGIAQRCLDFDVEDQHGHL 158

AtDME KAATQEKVKSKETGSAKKKNLKESATKKPANVGDMSNKSPEVTLKSCRKALNFDLENPGD 140

AtDML2 QEQSIFNNMQHNHQPDSDRRRLSLENLPGLYNMSCTQLLALAN-ATVATGSSIGASSSSL 87

AtDML3 KCDESAHLQDNSQTTHKKKEKKNSKEKHGIKHSESEHLQDDISQRVTGKGR--------- 74

AtROS1 ICPYTVEDQYHSSQLE-ERRFVGNKDMSGLDHLSFGDLLALANTASLIFSGQTPIPTRNT 86

:. .

HvDME HPGSQAQVVSCDPKEYQSSMPSTGQRNVQSQLTCHLDFT-TSSMYSSANQMADTQLLPAD 231

TaDME HPGSQAQVVSCDPKDYQPSMPSTGQRNAQSQLTCHLDFT-SSSMYSSANQMADTQLLPAD 231

BdDME HPGSQAQVVSTNLKDYQPSISSTSQGNVQSQLACHLGYTPTSSIYSSANQMADVHLLPAD 232

OsDME QSGSQVPVAEICTGPKRQSIPSTIQRDSQSQLACHVVSS--------------------- 215

SbDME DLVSQTRETEIQTGPGDTQPSISGVERSNVQVSCHWG----------------------- 195

ZmDME DLVSQTQETEIQTGPGDTQPSISGVERSNAQVSCHWGWG--------------------- 197

AtDME ARQGDSESEIVQNSSGANSFSEIRDAIGGTNGSFLDSVS--------------------- 179

AtDML2 SSQHPTDSWINSWKMDSNP----------------------------------------- 106

AtDML3 ----------RRNSKGT------------------------------------------- 81

AtROS1 EVMQKGTEEVESLSSVSNN----------------------------------------- 105

HvDME N--MKTSIYNSANQMANAQFLPAHNMPKGVLFDLNSS-TNQIQNEYANFLDGSAQFFQSG 288

TaDME N--MKTSIYSSANQMANAQFLPAHNMPKGVLFDLNSS-TNQIQNEYANFLDGPAQFFQSG 288

BdDME NNNMTEALYSSAKQMANAQLLPADNMLKGVSLDLNSS-TNQIQNEYANFVDRPTEFFQSG 291

OsDME ----TSSIHTSASQMVNAHLFPPDNMPNGVLLDLNNS-TSQLQNEHAKFVDSPARLFGSR 270

SbDME ---GTSSSISSVDPIVDIQGLRADCMPKSVNFDLNNSRVSQMPTNYSSLMDSSGQFFQYG 252

ZmDME ---GTSSSIISADPIVDIQGLQADCIPKRVNFDLNNSMASQMPTNYSSRMDSSGQFFQFG 254

AtDME ------------------------------QIDKTNGLGAMNQPLEVSMGNQPDKLSTGA 209

AtDML2 --------------------------------------------------------WTLS 110

AtDML3 -----------------------------------------------------------P 82

AtROS1 --------------------------------------------------------VAEQ 109

HvDME ITETLQTNPLLELCADMPDRNLPDLNSSITLMQGMPTNFTEYLLSSSQASVRETHMGKQM 348

TaDME ITETLQTNPLLELCAGMPDKNLPDLNSSITLMQGMPTNFTEYLLSSSQASVRETHMGKQM 348

BdDME IMETLRTGSLLELCSGMPDKNLPDLNRSIGLMQGMSTDCTDYFLSSSQASVKETKMARQM 351

OsDME IRQTSGTNSLLEIYAGMSDRNVPDLNSSISQTHSMSTDFAQYLLSSSQASVRETQMANQM 330

SbDME LREKVQTNQLLDSNSSLPVRHVSHLTSSVDHMRHPSANFDQYISKS-QDCTEKSPRHYQM 311

ZmDME LGEKVQTNQLLDYNCNLPARRVSHLSSSVDHMRHPLANFDQYISTS-QVCTENSRRHYQM 313

AtDME KLARDQQPDLLTRNQQCQFPVATQNTQFPMENQQAWLQMKNQLIGFPFGNQQPRMTIRNQ 269

AtDML2 KMQKQQYDVSTPQKFLCDLNLTPEELVSTSTQRTEPESPQITLKTPGKSLSETDHEPHDR 170

AtDML3 KKLRFNRPRILEDGKKPRNPATTRLRTISNKRRKKDIDSEDEVIPELATPTKESFPKRRK 142

AtROS1 ILKTPEKPKRKKHRPKVRREAKPKREPKPRAPRKSVVTDGQESKTPKRKYVRKKVEVSKD 169

. : .

HvDME PNCQRMPENPVTSAQCFEGVATRENFNLNSCLREGGATNQMCHGYRSTQNPIPPPNQIEG 408

TaDME PNCQRMPENPVTPAQCFEGVAARENFNLNSCLREGGVTNQICHGYRSSQSPIPPPKQIEG 408

BdDME LNCHRVPENPITHAQCSEGVAMRENSNSNVCSREAGVNNPMFYGYRSTQNPISPP----- 406

OsDME LNGHRMPENPITPSHCIERAALKEHLNHVPHAKAAVMNGQMPHGYRLAQNPILPPNHIEG 390

SbDME LSSYRISENMTAASQYTERVSMGGNFNPEACIGEGAIIKQMAQYYRLPESPFVPPKHNER 371

ZmDME PSSSRISENRTAASQYTERVSMGGNFNPGACIGEGTVIKQMAQCYRLPESPLVPPKHNER 373

AtDME QPCLAMGNQQPMYLIGTPRPALVSGNQQLGGPQGNKRPIFLNHQTCLPAGNQLYGSPTDM 329

AtDML2 IKKSVLGTGSPAAVKKR--KIARNDEKSQLETPTLKRK---------------------- 206

AtDML3 NEKIKRSVARTLNFKQEIVLSCLEFDKICG------------------------------ 172

AtROS1 QDATPVESSAAVETSTRPKRLCRRVLDFEAENGENQTNG--------------------- 208

.

HvDME HSAMENLNGLGRISDYLKFTSSPSPYRQTGGALGLHGSRSSSHMHALDTR-EHNASNSAY 467

TaDME HSAMENLNGLGTISDYLKFTTSPSPYRQTGGALGLHGSHSSSHVHALDTR-EHNASNGAH 467

BdDME --VMENLNELATINDYVKFTASS--YRPTGGAFGLHGPRDS----ALDNRGEHNASGGAH 458

OsDME YQVMENLSELVTTNDYLTASP----FSQTGAANRQHNIGDSMHIHALDPRRESNASSGSW 446

SbDME DVMNGNLNEFSVKNDYLKFSTNS--NYQTGAGFGFHDSPGYSDVLAMSKKREHNAISGHQ 429

ZmDME DVMNGNLNEFSVYNDYLNFSSNS--NYQTGAAFGFHDSPVYSDVLAMGKKREHNAISGHQ 431

AtDME HQLVMSTGGQQHGLLIKNQQPGS------------------------LIRGQQPCVPLID 365

AtDML2 ------------------------------------------------KIRPKVVREGKT 218

AtDML3 -----------------------------------------------------PIFPRGK 179

AtROS1 ------------------------------------------------DIREAGEMESAL 220

HvDME IPLGMNGDQ-QRNGWASVDACHAAPSQGSYFPETYKRLRTDNYSKYLNGAVGNTSTPSMY 526

TaDME IPLGMNLDQ-QRNGWASVDACHAAPSQGSYFPETYKRMRTDNYSKCLNGAVGNTSTPTMY 526

BdDME ISLGVKFDH-QRNGRASVGASHAATSQGSYFPATYKRMRMDNQSNCLNVDVANFSAPSGY 517

OsDME ISLGVNFNQ-QNNGWASAGAADAASSHAPYFSEPHKRMRTAYLNNYPNGVVGHFSTSSTD 505

SbDME ISFGIDFINSNRTRKFYSDDPFSTSSQTSYYPEACKRMRPEDHSNQLNGTTGKFSS-SLA 488

ZmDME ISFDIDFVNSNSTRKFCIDDLLSTSSQTSYYPEACKRMRPENHSNQLNVTTGKLSS-SSA 490

AtDME QQPATPKGFTHLNQMVATSMSSPGLRPHSQSQVPTTYLHVESVSRILNGTTGTCQRSRAP 425

AtDML2 KKASSKAG---IKKSSIAATATKTSEES----NYVRPKRLTRRSIRFDFDLQEEDEE--- 268

AtDML3 KRTTTRRRYDFLCFLLPMPVWKKQSRRSKRRKNMVRWARIASSSKLLEETLP-------- 231

AtROS1 QEKQLDSGNQELKDCLLSAPSTPKRKRSQGKRKGVQPKKNGSNLEEVDISMAQAAKRR-- 278

: :

HvDME LSNNQNTNVVSAINSNVFTLADAQRLIAREKSRASRGMINFGGTGYNTVKRPEMIEEHYI 586

TaDME LSNNRNTNVVSAINSNVFTLADAQRLIAREKSRASRGMISFGGSGYNIVKRPEMIKEHYR 586

BdDME LSNNRNTNVVSAINSNVFTLADAQRLIAREKLRASQGIIRFGASGNDMVKRPEMVRQDHR 577

OsDME LSNNENENVASAINSNVFTLADAQRLIAREKSRASQRMISFRSSKNDMVNRSEMVHQHGR 565

SbDME FSDGLNTNKVSAMNHGIGTLADIQRLMALEKSQASQQMINFITSQNNMN----------- 537

ZmDME FSGSLNTNNVSTINPGIGTLADIQRLMALEKSQASQQMIDFITSQNNMAG------ESTE 544

AtDME AYDSLQQDIHQGNKYILSHEISNGNGCKKALPQNSSLPTPIMAK--------------LE 471

AtDML2 ---------FCGIDFTSAGHVEGSSGE-------ENLTDTTLG----------------- 295

AtDML3 --------------LIVSHPTING------------------------------------ 241

AtROS1 -----QGPTCCDMNLSGIQYDEQCDYQKMHWLYSPNLQQGGMR----------------- 316

HvDME PAIHGTACSDSVEAPVKHFRHITEKITQVPSNPNTLQSQNYSPRIGSHQQQFWEGNTIEV 646

TaDME PAIHGTACSDSVEAPDKHFRHITEKITQVPSNPNTLQSQNCSPRIGSHQPQFWQGNTIEV 646

BdDME PAMHGTTYMDSAEASGRHFRFGTEEFTQLPSNPNSLQSQNYIPRIESHQLQPLEGNTVKG 637

OsDME PAPHGSACRESIEVPDKQFGLMTEELTQLPSMPNNPQREKYIPQTGSCQLQSLEHDMVKG 625

SbDME ------HDKGFIALPNKQFRSFIAQDIPLPGSTVNKLEENDILRNGVHQIHPWETTSRPH 591

ZmDME LAQQNNSDKGFVALHNKKFQSLTAQNIPLPDSTVNQSGESNILRNGIHQTQPWEITSRSH 604

AtDME EARGSKRQYHRAMGQTEKHDLNLAQQIAQSQDVERHNSSTCVEYLDAAKKTKIQKVVQEN 531

AtDML2 ------MFGHVPKGRRGQRR-------SNGFKKTDNDCLSSMLSLVNTGPGSF---MESE 339

AtDML3 -----------------------------QADASLHIDDTLVRHVVSKQTKKSANNVIEH 272

AtROS1 ------YDAICSKVFSGQQHNYVSAFHATCYSSTSQLSANRVLTVEERREGIFQGRQESE 370

.

HvDME SD-----LPVEQHNQSTAPQDDTRNSFCIGPSDQ---------LGGSINGDISRLPVTPT 692

TaDME SD-----LPVEQHNQSTAPQDDTRNSFCMGPSDE---------LGRSINGEISRFPVTPT 692

BdDME SD-----LPAELHKQSTSLQKDTRNIVCVDPSDE---------LGRRVNGERSRFSVPPT 683

OsDME HN-----LAGELHKQVTSPQVVIQSNFCVTPPDV---------LGRRTSGEHLRTLIAPT 671

SbDME HSSNNFALPNKLPGYLTAGN-AQLSSSTVNPSTENYIQSNAIHQHQCLENVVAKVPVLSE 650

ZmDME HSSDNFALPNKWSGYLTAGN-TQLSSITVNPSTENYIQSNAIYQYQCLENVVAKVPVLSG 663

AtDME LHG------------------------------------------------MPPEVIEIE 543

AtDML2 EDR------------------------------------------------PSDSQISLG 351

AtDML3 LN---------------------------------------------------------- 274

AtROS1 LNV------------------------------------------------LSDKIDTPI 382

HvDME GQSTGNDTLKKFGSQLETSGEVIMPLTSPR--NSSPRTDVLRNENHQVEVCGETTVAKPS 750

TaDME GQSTGNNTMEKFGFQLETSGEVIMPLTSPR--NSSPGTDVLRNENHQVEVCGETTVAKPS 750

BdDME TQSTSNDTAKKNSPQLS--GEVIRPLISPM--NPSPCTDVLSTESYQVEVCGETTAAKPS 739

OsDME HASTCKDTLKALSCQLESSRDIIRPPVNPI--GPSS-ADVPRTDNHQVKVSEETVTAKLP 728

SbDME THNISSQEGHNHCTAATTDEHIRTTSNEVVRSLTQEASQPTTNGNHNLNSSRVTAEAKST 710

ZmDME THNTSSQEGHNHSTAATN-EHIRTTSEEVVRSFTQRASEPTTHGNYNLNSSRVTAEANST 722

AtDME DDPTDGARKGKNTASISKGASKGNSSPVKKTAEKEKCIVPKTPAKKGRAGRKKSVPPPAH 603

AtDML2 RQRSIMATRPRNFRSLKKLLQRIIPSKRDRKGCKLPRGLPKLTVASKLQLKVFRKKRSQR 411

AtDML3 --------------------------------------RQITYQKDHGLSSLADVPLHIE 296

AtROS1 KKKTTGHARFRNLSSMNKLVE--VPEHLTSGYCSKPQQNNKILVDTR--VTVSKKKPTKS 438

HvDME EK-RKAGRPRKEIKPGENPKPRGRPRKEKVVGAELASK-GSHTDPLPNVDISVISGPHAG 808

TaDME EK-RKAGRPRKEIKPGENPKPRGRPRKQKVVGAELASK-GSHTDPWPNEDISVISGPHAG 808

BdDME EK-RKVGRPRKEIKPGVKPKPRGRPRKEKLLGTELKS---SHTDPLQIGDISFVSGPHVR 795

OsDME EK-RKVGRPRKELKPGEKPKPRGRPRKGKVVGGELASK-DSHTNPLQNESTSCSYGPYAG 786

SbDME EKPRKRGRPRKVVNPNGEPKERVTKGKQNVGHAKPISPKGSCTDFLKTNGITYASEPSTG 770

ZmDME EKPRKRGRPRKVVKPNGEPKERGTKGKQNVSHEKPTSQKGSHTDILKTNGISYASEPSTG 782

AtDME ASEIQLWQPTPPKTPLSRSKPKGKGRKSIQDSGKARGPSGELLCQDSIAEIIYRMQNLYL 663

AtDML2 NRVASQFNARILDLQWRRQNPTGTSLADIWERSLTIDAITKLFEELDINKEG---LCLPH 468

AtDML3 DTLIKSASSVLSERPIKKTK--------------------------DIAKLIKDMGRLKI 330

AtROS1 EKSQTKQKNLLPNLCRFPPSFTGLSPDELWKRRNSIETISELLRLLDINREHSETALVPY 498

.

HvDME ESPGPKGINTERS-------GESFPGSIAPPVDPLDLIIQKIKVLDINKSDGIGSAEPHG 861

TaDME VSPGSKGINTERS-------GESFPGAIAPPLDPLDLIIQKIQVLDINKSDDTGSAEPHG 861

BdDME ESLAPKGVNTERS-------GESFLRNIESLADPLDLIIQKIKVLDINKSDDTGAVELHS 848

OsDME EASVGRAVKANRV-------GENISGAMVSLLDSLDIVIQKIKVLDINKSEDPVTAEPHG 839

SbDME ITPRMSTVESKSSDQDKEIHGGVIPQAAAASVDPLDGIIQKIKLLSINGPDKIVAEVPKN 830

ZmDME ITPRMAIVESKSSDQDKEIHGGVIPQATAISVDLLDGIIQKIKLLSISRPDNVVAEIPKD 842

AtDME GDKEREQEQNAMV----------------------------------------------- 676

AtDML2 NRETALILYKKS------------------------------------------------ 480

AtDML3 NKKVT------------------------------------------------------- 335

AtROS1 TMNSQIVLFGGG------------------------------------------------ 510

**A**

HvDME ALVPYKGEFGAIIPYEGKGKRKYARAKVNLDPVTALMWKLLMEPDM-VDGSEGMDKDKEK 920

TaDME ALVPYKGEFGAIIPYEGKGKRKYARAKVNLDPVTALMWKLLMEPDM-VDGSEGMDKDKEK 920

BdDME ALVPYKGEVGAVVPYEGKVKRKRARAKVSLDPVTALMWKLLMEPDM-VDGSEEMDKDKEK 907

OsDME ALVPYNGEFGPIVPFEGKVKRKRSRAKVDLDPVTALMWKLLMGPDM-SDCAEGMDKDKEK 898

SbDME ALVPYEGEFGALVAFEGKTKKSRSRAKVNIDPVTTMMWNLLMGPDM-GDGAEGLDKDKEK 889

ZmDME ALVPYEGEFGALVAFEGKTKKNRSRAKVNIDPVTTMMWNLLMGPDM-GDGAEGLDKDKEK 901

AtDME ------LYKGDGALVPYESKKRKPRPKVDIDDETTRIWNLLMGKGDEKEGDEEKDKKKEK 730

AtDML2 ---------YEEQKAIVKYSK-KQKPKVQLDPETSRVWKLLMSSID-CDGVDGSDEEKRK 529

AtDML3 --------------TMIKADKKLVTAKVNLDPETIKEWDVLMVNDSPSRSYD--DKETEA 379

AtROS1 ---------AGAIVPVTPVKKPRPRPKVDLDDETDRVWKLLLENIN-SEGVDGSDEQKAK 560

.: .**.:* * *.:*: : *:..

HvDME WLDEERKIFRGRIDSFIARMHLVQGDRRFSPWKGSVVDSVVGVFLTQNVSDHLSSSAFMA 980

TaDME WLEEERKIFRGRIDSFIARMHLVQGDRRFSPWKGSVVDSVVGVFLTQNVSDHLSSSAFMA 980

BdDME WLDEERKIFQGRVDSFIARMHLVQGDRRFSPWKGSVVDSVVGVFLTQNVSDHLSSSAFMA 967

OsDME WLNEERKIFQGRVDSFIARMHLVQGDRRFSPWKGSVVDSVVGVFLTQNVSDHLSSSAFMA 958

SbDME WLDEERRVFRGRVDSFIARMHLVQGDRRFSRWKGSVVDSVVGVFLTQNVSDHLSSSAFMA 949

ZmDME WLDEERKVFRGRVDSFIARMHLVQGDRRFSRWKGSVVDSVVGVFLTQNVSDHLSSSAFMA 961

AtDME WWEEERRVFRGRADSFIARMHLVQGDRRFSPWKGSVVDSVIGVFLTQNVSDHLSSSAFMS 790

AtDML2 WWEEERNMFHGRANSFIARMRVVQGNRTFSPWKGSVVDSVVGVFLTQNVADHSSSSAYMD 589

AtDML3 KWKKEREIFQTRIDLFINRMHRLQGNRKFKQWKGSVVDSVVGVFLTQNTTDYLSSNAFMS 439

AtROS1 WWEEERNVFRGRADSFIARMHLVQGDRRFTPWKGSVVDSVVGVFLTQNVSDHLSSSAFMS 620

.:**.:*: * : ** **: :**:* *. *********:*******.:*: **.*:*

HvDME LAAKFPAKPEVSKISADRMFHTASENVG---CSGLFGDSVKLPGGILVEEASNTTGSLVT 1037

TaDME LAAKFPAKPEVSKISADRMFHTASENVG---CSGLFGDSVKLPGGILVEEASNTTGSLVT 1037

BdDME LAAKFPAKSEVSKIPSDRMFHTPSEKNGG--CSGLFGDSVKLQGNILVEEVSNTTGSLVT 1025

OsDME LAAKFPVKPEASEKPANVMFHTISENGD---CSGLFGNSVKLQGEILVQEASNTAASFIT 1015

SbDME VAAKFPAKTEVPEKPVAEMSHTPPEQKD--SCSGLFGDSIKLQGKIFIEEVSDVR-SLIT 1006

ZmDME VAAKFPAKPEVPEKPVAEMSHTP-EQKDSCSCSGLFGDSIKLQGKMFIEEISDVR-SLIT 1019

AtDME LAARFPPKLSSSREDERNVRSVVVEDPEGCILNLNEIPSWQEKVQHPSDMEVSGVDSGSK 850

AtDML2 LAAEFPVEWNFNKGSCHEEWGSSVTQETILNLDPRTGVS-TPRIRNPTRVIIEEIDDDEN 648

AtDML3 VAAKFP-------VDAREGLSYYIEEPQ-------------------------------- 460

AtROS1 LASQFPVP--FVPSSNFDAGTSSMPSIQITYLDSEETMS-SPPDHNHSSVTLKNTQPDE- 676

:*:.** .

HvDME TEEKEGSNSSGLFGNSPGDGVDCTAGVYCNSYGTLPVRLHE-----GKTPAVGTESVVEV 1092

TaDME TEEKEGSNSSGLFGNSPGDGVDCTAGVYYNSYGTLPVRLHE-----GKTPAVGTESVVEV 1092

BdDME TEEKEGSNSIGLFGNSPGGGVDCAAGVYYNSCGMLPVRLPE-----SKPPSVGTGSFVEV 1080

OsDME TEDKEGSNSVELLGSSFGDGVDGAAGVYSNIYENLPARLHA-----TRRPVVQTGNAVEA 1070

SbDME TEDNEESNSNELIGSSAGYGINRATGGCHVSYRKSLTGSHGNGLSGSVFPTTGFSSVVET 1066

ZmDME TEDNEESNSNELIGSSAGYGVNHATGGCHVSYRKSLTESHENGLSGSVFPTTGFSSVVET 1079

AtDME EQLRDCSNSGIERFNFLEKSIQNLEEEVLSSQDSFDPAIFQ-----------SCGRVGSC 899

AtDML2 DIDAVCSQESSKTSDSSITSADQSKTMLLDPFNTVLMN----------------EQVDSQ 692

AtDML3 ------------------------------------------------------------

AtROS1 EKDYVPSNETSRSSSEIAISAHESVDKTTDSKEYVDSDRKG-----------SSVEVDKT 725

HvDME EDGALEDVVSSQNSAISSQSSPDYLFHMTDHMFPSTLLNFTAEDFVGRNMANGTSNSTTY 1152

TaDME EDGALEDVVSSQNSAISSQSSPDYLFHMTDHMFPSTLLNFTAEDFVGRNMANGTSNSTTY 1152

BdDME DDGALEDVVSSQNSTISSQSSPDYLLHVTDPMFPSRLLNFTVEDFVGRNMAHGTSNSTTY 1140

OsDME EDGSLEGVVSSENSTISSQNSSDYLFHMSDHMFSSMLLNFTAEDIGSRNMPKATR--TTY 1128

SbDME EDGSLEDVISSQNSAVSSQNSSDYLFHRTDPTGSSSLQNFTEEGCIMRNISSGTGRSTDY 1126

ZmDME EDGSLEDVISSQNSAVSSQNSPDYLFHRTDPIGSSSLQNFTEEGYIMRNISNGTGSSTDC 1139

AtDME SCSKSDAEFPTTRCETKTVSGTSQSVQTGSPNLSDEICLQGNERPHLYEGSGDVQKQETT 959

AtDML2 MVKGKGHIPYTDDLNDLSQGISMVSSASTHCELN--LNEVPPEVELCS----HQQDPES- 745

AtDML3 ------------------------------------------------------------

AtROS1 DEKCRVLNLFPSEDSALTCQHSMVSDAPQNTERAGSSSEIDLEGEYRTSFMKLLQGVQV- 784

HvDME TELLKMQELKSKPSEKE----YDGVPIQCTNRGSIPSEVHNLSSKAQPLHASGSYHQNVR 1208

TaDME TELLKMQELKSKPNEKE----YDGVPIQCTNRGSIPSEVHNLNSKTQPLHASGSYHQNGR 1208

BdDME TELLKMQELKSKPNENSGLL-KYGAQIQVTNKRSVLNEVRNPSLKHQPLHSSVSYHQNGQ 1199

OsDME TELLRMQELKNKSNETIESSEYHGVPVSCSNNIQVLNGIQNIGSKHQPLHSSISYHQTGQ 1188

SbDME TAFLPIQDPTGMLGLSEYYG-LNPLPVSGVNKG----VLLDLNRSYQPLHTSMPYVQNSE 1181

ZmDME SGFLPIQDPKGTLGLSEYYG-HNPLLVSGVNKG----VLLDLNRSYQPLHTSMPYVQNSE 1194

AtDME NVAQKKPDLEKTMNWKDSVCFGQPRNDTNWQTTPSSSYEQCATRQPHVLDIEDFGMQGEG 1019

AtDML2 ------------------------------------------------------------

AtDML3 ------------------------------------------------------------

AtROS1 ------------------------------------------------------------

HvDME AHLPDMTFGSDLERSVYTGLNRTDDSRVSPAEIRYDCSLSSPGIDSENKAQTTDSLTALL 1268

TaDME AHLPDITFSSDLEHSVYTGLNRTDDSRVTPAEIRYDCSLSSPGIDSENRSQTTDSLTALL 1268

BdDME AHLPDITYANDLEHSVYPGINRIDDSSGTVAPARFDCPLPSPGTDSENKTKMTDSLTALL 1259

OsDME VHLPDIVHASDLEQSVYTGLNRVLDSNVTQTSY---YPSPHPGIACNNETQKADSLSNML 1245

SbDME SDFTGVSCFSHMDKSFHTGPDRVNLSSVTQSEAS-------------------------L 1216

ZmDME SDFTGVSCFSHMDKSFHTGPNRVNLSSVTQSEAS-------------------------L 1229

AtDME LGYSWMSISPRVDRVKNKNVPRRFFRQ--------------------------------- 1046

AtDML2 ------------------------------------------------------------

AtDML3 ------------------------------------------------------------

AtROS1 ------------------------------------------------------------

HvDME YGIDGSLSQDKIPYPSMATRGADSISILMDKYFYP-SSSETVSFGREQLSCENNLQRNDV 1327

TaDME YGIDGSLSQDKIPFPSMATQGADSISTLMDKYFHP-SSSETASFAREQLSCENNLQRNDV 1327

BdDME YCIDESLSHNKISFPYGTTPGADLSSPMIDKYFQP-ASADTVSFTREQ-SYEKNLSRNGI 1317

OsDME YGIDRSDKTTSLSEP----------TPRIDNCFQP-LSSEKMSFAREQSSSENYLSRNEA 1294

SbDME YPTDPLQQGD--------------FSPVIKQNFQP-HSSDKVPFFKEHSSCGNDFSRNKT 1261

ZmDME YPTDPLQQDE--------------FSPVIKQNFQPLYSSDKVSLFKEHCSYGNDFSRNKT 1275

AtDME ------------------------GGSVPREFTGQIIPSTPHELPGMGLSGSSSAVQEHQ 1082

AtDML2 ------------------------------------------------------------

AtDML3 ------------------------------------------------------------

AtROS1 ------------------------------------------------------------

HvDME VAAFAKQHETLNLQEECTARAKQIGGENYQSGCSQQYGNVGLSSNMDGSHCSSNLYQNEK 1387

TaDME VAAFVKQHGTLNLQEECTARAKQIGGENCQSGCSQQYGNVGLSSNMDGSHCSSNLYQNEK 1387

BdDME EAALVEQHDTLNLQEECTTRANQIGGDNHQSVCSQQYGNVGLPTNKDESRYSSNLCQNEK 1377

OsDME EAAFVKQHGTSNVQGDNTVRTEQNGGENSQSGYSQQDDNVGFQTATTSNLYSSNLCQNQK 1354

SbDME ETPSVEPLVYSNPQEVYTTSTDPMGAEQFQSGCGQQDNDARIQTASHERHQSSALCENQN 1321

ZmDME EAAIMEPLVYSNPQELYTTSTEQMGVEQFQSGCGQQDNDVRVQTTSYERHQSSTLCGNQN 1335

AtDME DDTQHNQQDEMNKASHLQKTFLDLLNSSEECLTRQSSTKQNITDGCLPRDRTAEDVVDPL 1142

AtDML2 -----------------------------------TIQTQDQQESTR-----TEDVK--K 763

AtDML3 ------------------------------------------------------------

AtROS1 -----------------------------------SLEDSNQVSPNMSPGDCSSEIKGFQ 809

HvDME ANSELLQRVASDSIEKPKDTNKALPEVPAD-RSKTKKTRAG--KKRTYDWDILRKEVLAN 1444

TaDME ANSELLERVASESIEKPKDTNKALPEVPAD-RSKAKKARAG--KKRTYDWDILRKEVLAS 1444

BdDME ANSEPLQGVALDSIEKLKDIRKSFPEVPADGSSKAKKARVGTGKKRAYDWDILRKEVLVN 1437

OsDME ANSEVLHGVSSNLIENSKDDKKTSPKVPVD-GSKAKRPRVGAGKKKTYDWDMLRKEVLYS 1413

SbDME SHSEVLQGVAAGSTQKFIDIQKGPPEAQQN-GSKAKKVRGR-PR-KTYDWDSLRKEVLSN 1378

ZmDME SQLEILQGVASGSTQKFIDTQKSPSEVQQN-GSKAKKVRGR-PKTKTYDWDSLRKEVFSN 1393

AtDME SNNSSLQNILVESNSSNKEQTAVEYKETNATILREMKGTLADGKKPTSQWDSLRKDVEGN 1202

AtDML2 NRKKPTTS---------------------KPKKKSK-ESAKSTQKKSVDWDSLRKEAESG 801

AtDML3 -DAKSSECIILSDES----ISKVEDHENTAKRKNEKTGIIED---EIVDWNNLRRMYTKE 512

AtROS1 SMKEPTKSSVDSSEPGCCSQQDGDVLSCQKPTLKEKGKKVLKEEKKAFDWDCLRREAQAR 869

. . :*: **:

**helix**

**helix**

**helix**

HvDME RGNEERSENAKDALDWETIRQINVKEISNTIRERGMNNMLAERIKDFLNRVVRDHGSIDL 1504

TaDME RGNEERGENAKDALDWETIRQIDVKEISNAIRERGMNNMLSERIQDFLNRVVRDHGSIDL 1504

BdDME HGNEERANNAKDALDWETIRQIDVKEISDTIRERGMNNMLAERIKAFLNRLVTDHGSIDL 1497

OsDME HGNKERSQNAKDSIDWETIRQAEVKEISDTIRERGMNNMLAERIKDFLNRLVRDHGSIDL 1473

SbDME GGDKQRSHDARDTVDWEAVRQAEVREISETIRERGMNNMLAERIKEFLNRLVTDHGSIDL 1438

ZmDME GGDKQRNNDARDTVDWEAVRQAEVREISETIRERGMNNMLAERIKEFLNRLVTDHGGIDL 1453

AtDME EGRQERNKNNMDSIDYEAIRRASISEISEAIKERGMNNMLAVRIKDFLERIVKDHGGIDL 1262

AtDML2 GRKRERTERTMDTVDWDALRCTDVHKIANIIIKRGMNNMLAERIKAFLNRLVKKHGSIDL 861

AtDML3 GSRPE---MHMDSVNWSDVRLSGQNVLETTIKKRGQFRILSERILKFLNDEVNQNGNIDL 569

AtROS1 AGIREKTRSTMDTVDWKAIRAADVKEVAETIKSRGMNHKLAERIQGFLDRLVNDHGSIDL 929

: *::::. :* : * .** . *: ** **: * .:*.***

**(GPD) gly/pro-rich region**

**with conserved apartate**

**Helix- hairpin- helix**

helix


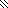

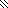


HvDME EWLRHVDPDKAKEYLLSIRGLGLKSVECVRLLTLHHMAFPV**D**TNVGRICVRLGWVPLQPL 1564

TaDME EWLRYVDPDKAKEYLLSIRGLGLKSVECVRLLTLHHMAFPV**D**TNVGRICVRLGWVPLQPL 1564

BdDME EWLRYVDPDKAKEYLLSIRGLGLKSVECVRLLTLHHMAFPV**D**TNVGRICVRLGWVPLQPL 1557

OsDME EWLRYVDSDKAKDYLLSIRGLGLKSVECVRLLTLHHMAFPV**D**TNVGRICVRLGWVPLQPL 1533

SbDME EWLRDVQPDKAKDFLLSIRGLGLKSVECVRLLTLHHMAFPV**D**TNVGRICVRLGWVPLQPL 1498

ZmDME EWLRDVPPDKAKDFLLSIRGLGLKSVECVRLLTLHHMAFPV**D**TNVGRICVRLGWVPLQPL 1513

AtDME EWLRESPPDKAKDYLLSIRGLGLKSVECVRLLTLHNLAFPV**D**TNVGRIAVRMGWVPLQPL 1322

AtDML2 EWLRDVPPDKAKEYLLSINGLGLKSVECVRLLSLHQIAFPV**D**TNVGRIAVRLGWVPLQPL 921

AtDML3 EWLRNAPSHLVKRYLLEIEGIGLKSAECVRLLGLKHHAFPV**D**TNVGRIAVRLGLVPLEPL 629

AtROS1 EWLRDVPPDKAKEYLLSFNGLGLKSVECVRLLTLHHLAFPV**D**TNVGRIAVRLGWVPLQPL 989

**** .. .* :**.:.*:****.****** *:: ***********.**:* ***:**

**4Fe-4S binding**

βββββββ

βββββββ

helix

helix

HvDME PESLQLHLLELYPMLENIQKYLWPRLCKLDQRTLYELHYQMITFGKVFCTKSKPNCNACP 1624

TaDME PESLQLHLLELYPMLENIQKYLWPRLCKLDQRTLYELHYQMITFGKVFCTKSKPNCNACP 1624

BdDME PESLQLHLLELYPMLENIQKYLWPRLCKLDQRTLYELHYQMITFGKVFCTKSKPNCNACP 1617

OsDME PESLQLHLLEMYPMLENIQKYLWPRLCKLDQRTLYELHYQMITFGKVFCTKSKPNCNACP 1593

SbDME PESLQLHLLEMY-----------------------ELHYQMITFGKVFCTKSKPNCNSCP 1535

ZmDME PESLQLHLLEMY-----------------------ELHYQMITFGKVFCTKSKPNCNSCP 1550

AtDME PESLQLHLLELYPVLESIQKFLWPRLCKLDQRTLYELHYQLITFGKVFCTKSRPNCNACP 1382

AtDML2 PDELQMHLLELYPVLESVQKYLWPRLCKLDQKTLYELHYHMITFGKVFCTKVKPNCNACP 981

AtDML3 PNGVQMHQLFEYPSMDSIQKYLWPRLCKLPQETLYELHYQMITFGKVFCTKTIPNCNACP 689

AtROS1 PESLQLHLLEMYPMLESIQKYLWPRLCKLDQKTLYELHYQMITFGKVFCTKSKPNCNACP 1049

*: :*:* * * ****::********** ****:**

HvDME MRAECKHFASAFASARLALPGPEEKSLVTSGNPIASGSCQQPYISPMRLN-QLDWNAHAH 1683

TaDME MRAECKHFASAFASARLALPGPEEKSLVTSGNPIASGSCQQPYISSMRLN-QLDWNANAH 1683

BdDME MRAECKHFASAFASARLALPGPEEKSLVTSGNPIVAESCQQPYISSRPLN-QLDWNAHPH 1676

OsDME MRAECKHFASAFASARLALPGPEEKSLVTSGTPIAAETFHQTYISSRPVVSQLEWNSNTC 1653

SbDME MRAECKHFASAFASARLALPAPEEKRLATSEDANVVEFCHQTYINSGTVG-QLEWSANYP 1594

ZmDME LRAECKHFASAFASARLALPAPEEKRLATSEDPNVVEFCHQTYINSGAVG-ELEWSANYP 1609

AtDME MRGECRHFASAYASARLALPAPEERSLTSATIPVPPESYPPVAIPMIELPLPLEKSLASG 1442

AtDML2 MKAECRHYSSARASARLALPEPEESDRTSVMIHERRSKRKPVVVNFRPSLFLYQEKEQE- 1040

AtDML3 MKSECKYFASAYVSSKVLLESPEEKMHEPNTFMN--AHSQDVAVDMTSNINLVEECVSSG 747

AtROS1 MKGECRHFASAFASARLALPSTEKGMGTPDKNPLPLHLPEPFQREQGSEVVQHSEP---- 1105

::.**::::** .*::: * .*: . .

HvDME DHILD-NCQPIIEEPASPEPEPETAEMRESAIED-------IFLDDPEEIPTIKLNFEEF 1735

TaDME DHILD-NRQPIIEEPASPEPEPETAEMRESAIED-------IFLDDPEEIPTIKLNFEEF 1735

BdDME DHVLG-NRQPIIEEPASPEPEPETAELKEGAIED-------MFFDDPEEIPTIKLNFEEF 1728

OsDME HHGMN-NRQPIIEEPASPEPEHETEEMKECAIED-------SFVDDPEEIPTIKLNFEEF 1705

SbDME KHAVSGNHQPIIEEPLSPEPEPENVEAKEGAIED-------FFCEDPDEIPTINLNIEEF 1647

ZmDME KHAVCGNLQPFIEEPLSPEPEPENVEAKDGAIED-------FFNEDPDEIPTINLNIEEF 1662

AtDME APSNRENCEPIIEEPASPGQECTEIT-ESDIED-------AYYNEDPDEIPTIKLNIEQF 1494

AtDML2 -AQRSQNCEPIIEEPASPEPEYIEHDIEDYPRDKNNVGTSEDPWENKDVIPTIILNKEAG 1099

AtDML3 CSDQAICYKPLVEFPSSPRAEIPESTDIEDVPF-------MNLYQSYASVPKIDFDLDAL 800

AtROS1 -AKKVTCCEPIIEEPASPEPETAEVSIAD---------IEEAFFEDPEEIPTIRLNMDAF 1155

:*::* * ** * . :. :*.* :: :

HvDME AQNLKNYMQVN-NIEMEDADMSSALVAITPEAASIPTP---RLKNVSRLRTEHQVYELPD 1791

TaDME AQNLKNYMQVN-NIEMEDADMSSALVAITPEAASIPTP---RLKNVSRLRTEHQVYELPD 1791

BdDME AQNLKNYMQVN-NIDIEDADMSSALVAITPEAASIPTP---RLKNVSRLRTEHQVYELPD 1784

OsDME TQNLKSYMQAN-NIEIEDADMSKALVAITPEVASIPTP---KLKNVSRLRTEHQVYELPD 1761

SbDME TQNLKNYMQAN-NVDIEYADMSKALVAITPDAASIPTP---KLKNISRLRTEHQVYELPD 1703

ZmDME TQNLKNYMQAN-HVEIEYADMSKALVAITPEAASIPTP---KLKNVSRLRTEHQVYELPD 1718

AtDME GMTLREHMERN--MELQEGDMSKALVALHPTTTSIPTP---KLKNISRLRTEHQVYELPD 1549

AtDML2 TS---HDLVVN--KEAG---TSHDLVVLSTYAAAIPRR---KLKIKEKLRTEHHVFELPD 1148

AtDML3 KKSVEDALVISGRMSSSDEEISKALVIPTPENACIPIKPPRKMKYYNRLRTEHVVYVLPD 860

AtROS1 TSNLKKIMEHN--KELQDGNMSSALVALTAETASLPMP---KLKNISQLRTEHRVYELPD 1210

: . . * ** . :.:* ::* .:***** *: ***

**B**

HvDME SHPLLEGYDQREPDDPCPYLLSIWTPGETAQSIDAPKTAC-NSNESGKLCDSSACFSCNS 1850

TaDME SHPLLEGYDQREPDDPCPYLLSIWTPGETAQSIDAPKTAC-NSNESGKLCDSSACFSCNS 1850

BdDME SHPLLEGFDQREPDDPCPYLLSIWTPGETAQSADAPMTSC-NSHESGKLCDSSACFSCNS 1843

OsDME SHPLLEGFNQREPDDPCPYLLSIWTPGETAQSTDAPKSVC-NSQENGELCASNTCFSCNS 1820

SbDME SHPLLDGFEQREPDDPCPYLLSIWTPGETAQSTDAPKTFC-DSGETGRLCGSSTCFSCNS 1762

ZmDME SHPLLEGFEQREPDDPCPYLLSIWTPGETAQSTNAPKTFC-DSGETGQLCGSLTCFSCNS 1777

AtDME SHRLLDGMDKREPDDPSPYLLAIWTPGETANSAQPPEQKC-GGKASGKMCFDETCSECNS 1608

AtDML2 HHSILEGFERREAEDIVPYLLAIWTPGETVNSIQPPKQRCALFESNNTLCNENKCFQCNK 1208

AtDML3 NHELLHDFERRKLDDPSPYLLAIWQPGETSSSFVPPKKKC-SSDGS-KLCKIKNCSYCWT 918

AtROS1 EHPLLAQLEKREPDDPCSYLLAIWTPGETADSIQPSVSTC-IFQANGMLCDEETCFSCNS 1269

* :* ::*: :* .***:** **** .* .. * . :* * * .

HvDME MREAQAQTVRGTILVPCRTAMRGSFPLNGTYFQVNEVFADHDSSRNPVDVPRRWIWDLPR 1910

TaDME MREAQAQTVRGTILVPCRTAMRGSFPLNGTYFQVNEVFADHDSSRNPVDVPRRWIWDLPR 1910

BdDME IREVQAQKVRGTLLIPCRTAMRGSFPLNGTYFQVNEVFADHDSSRNPIDVPRSWIWNLPR 1903

OsDME IREAQAQKVRGTLLIPCRTAMRGSFPLNGTYFQVNEVFADHDSSRNPIDVPRSWIWNLPR 1880

SbDME IREMQAQKVRGTLLIPCRTAMRGSFPLNGTYFQVNEVFADHYSSQNPIDVPRSWIWDLPR 1822

ZmDME LREMQAQKVRGTLLIPCRTAMRGSFPLNGTYFQVNEVFADHCSSQNPIDVPRSWIWDLPR 1837

AtDME LREANSQTVRGTLLIPCRTAMRGSFPLNGTYFQVNELFADHESSLKPIDVPRDWIWDLPR 1668

AtDML2 TREEESQTVRGTILIPCRTAMRGGFPLNGTYFQTNEVFADHDSSINPIDVPTELIWDLKR 1268

AtDML3 IREQNSNIFRGTILIPCRTAMRGAFPLNGTYFQTNEVFADHETSLNPIVFRRELCKGLEK 978

AtROS1 IKETRSQIVRGTILIPCRTAMRGSFPLNGTYFQVNEVFADHASSLNPINVPRELIWELPR 1329

:* .:: .***:*:********.*********.**:**** :* :*: . * :

HvDME RTVYFGTSVPSIFKGLTTEDIQQCFWRGFVCVRGFDRTSRAPRPLYARLHFPASKIIRNK 1970

TaDME RTVYFGTSVPSIFKGLTTEDIQQCFWRGFVCVRGFDRTSRAPRPLYARLHFPASKITRNK 1970

BdDME RTVYFGTSVPTIFKGLTTEDIQHCFWRGFVCVRGFDRISRAPRPLYARLHFPASKITRNK 1963

OsDME RTVYFGTSIPTIFKGLTTEEIQHCFWRGFVCVRGFDRTSRAPRPLYARLHFPASKITRNK 1940

SbDME RTVYFGTSVPTIFRGLTTEQIQQCFWRGFVCVRGFDRTVRAPRPLYARLHFPASKVVRGK 1882

ZmDME RTVYFGTSVPTIFRGLTTEEIQQCFWRGFVCVRGFDRTVRAPRPLYARLHFPASKVVRGK 1897

AtDME RTVYFGTSVTSIFRGLSTEQIQFCFWKGFVCVRGFEQKTRAPRPLMARLHFPASKLKNNK 1728

AtDML2 RVAYLGSSVSSICKGLSVEAIKYNFQEGYVCVRGFDRENRKPKSLVKRLHCSHVAIRTKE 1328

AtDML3 RALYCGSTVTSIFKLLDTRRIELCFWTGFLCLRAFDRKQRDPKELVRRLHTPPDERGPKF 1038

AtROS1 RTVYFGTSVPTIFKGLSTEKIQACFWKGYVCVRGFDRKTRGPKPLIARLHFPASKLKGQQ 1389

*. * *:::.:* : * .. *: * *::*:*.*:: * *: * *** .

HvDME KGAASV-GIDDA 1981

TaDME KGAASA-GTDDA 1981

BdDME KPTASA-ARDDA 1974

OsDME KSAGSAPGRDDE 1952

SbDME KPGAAR--EEE- 1891

ZmDME KPGAAS--VEE- 1906

AtDME T----------- 1729

AtDML2 KTEE-------- 1332

AtDML3 MSDDDI------ 1044

AtROS1 ANLA-------- 1393
