# Supplementary figures and images for: The study of a barley epigenetic regulator, HvDME, in seed development and under drought
Source: BMC Plant Biol. 2013 Oct 31;13:172. doi: 10.1186/1471-2229-13-172 (PMC4228467; doi:10.1186/1471-2229-13-172)

**Additional File 4**

**Genomic organization of cereal *DME* genes**


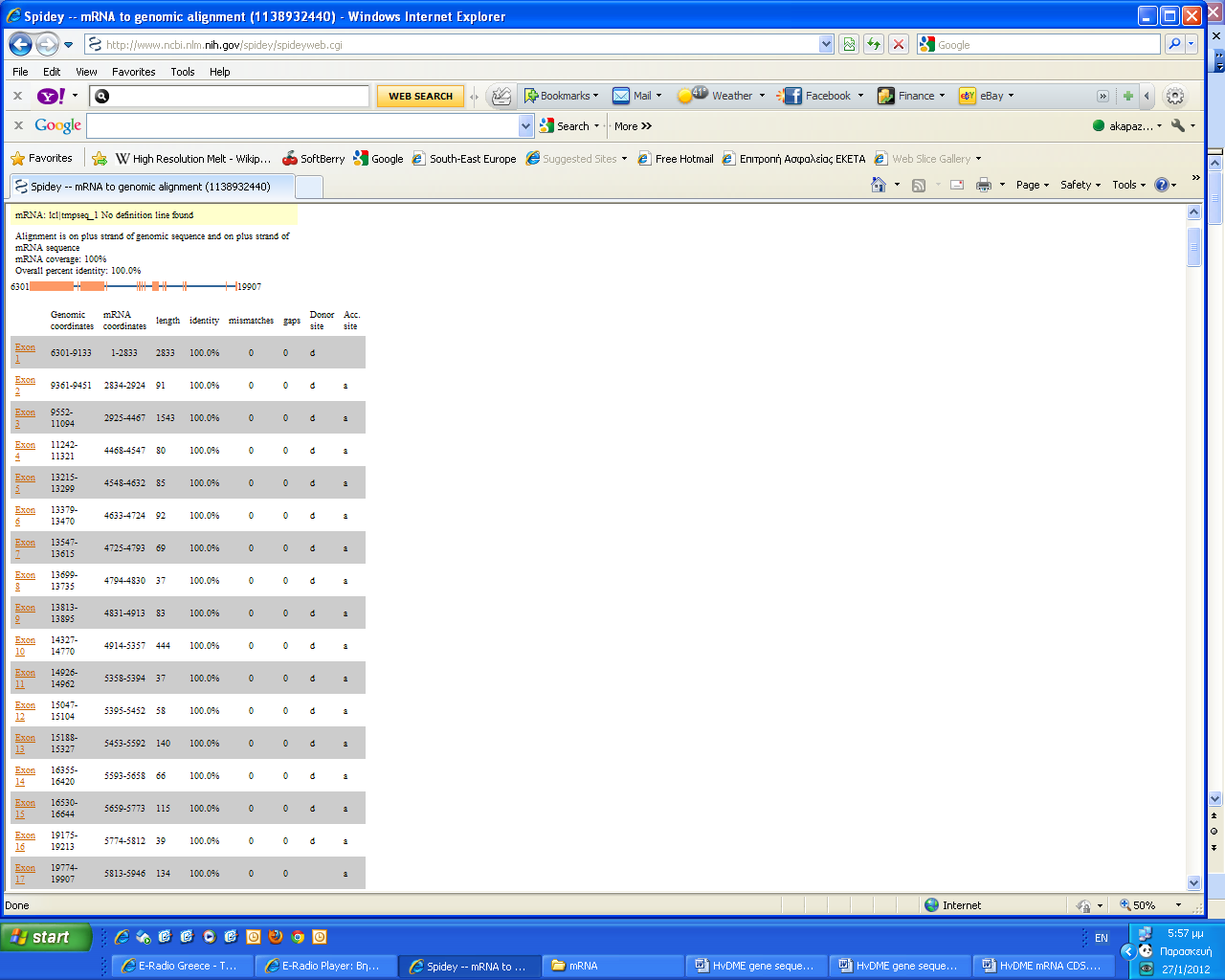


**HvDME**

**BdDME**


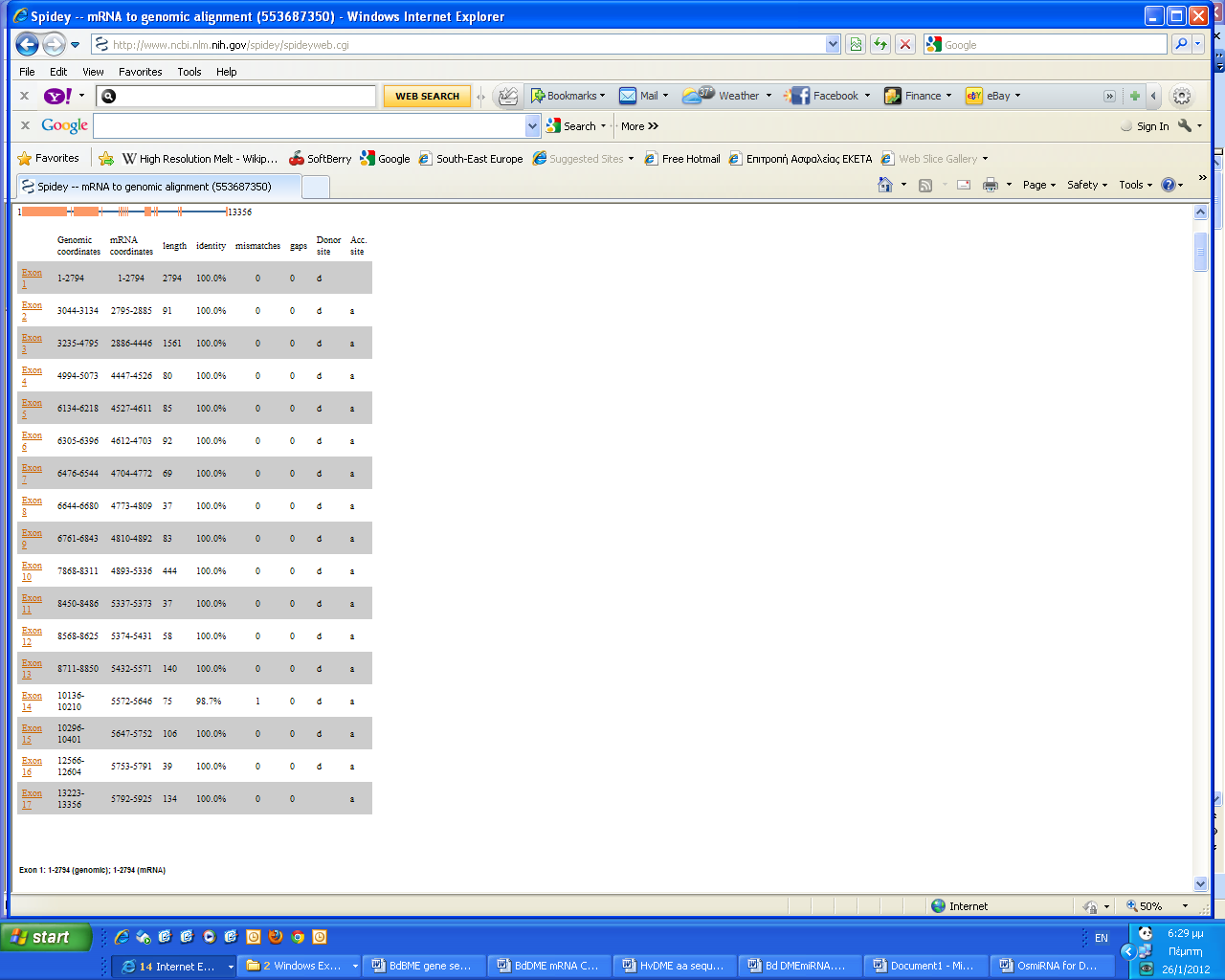

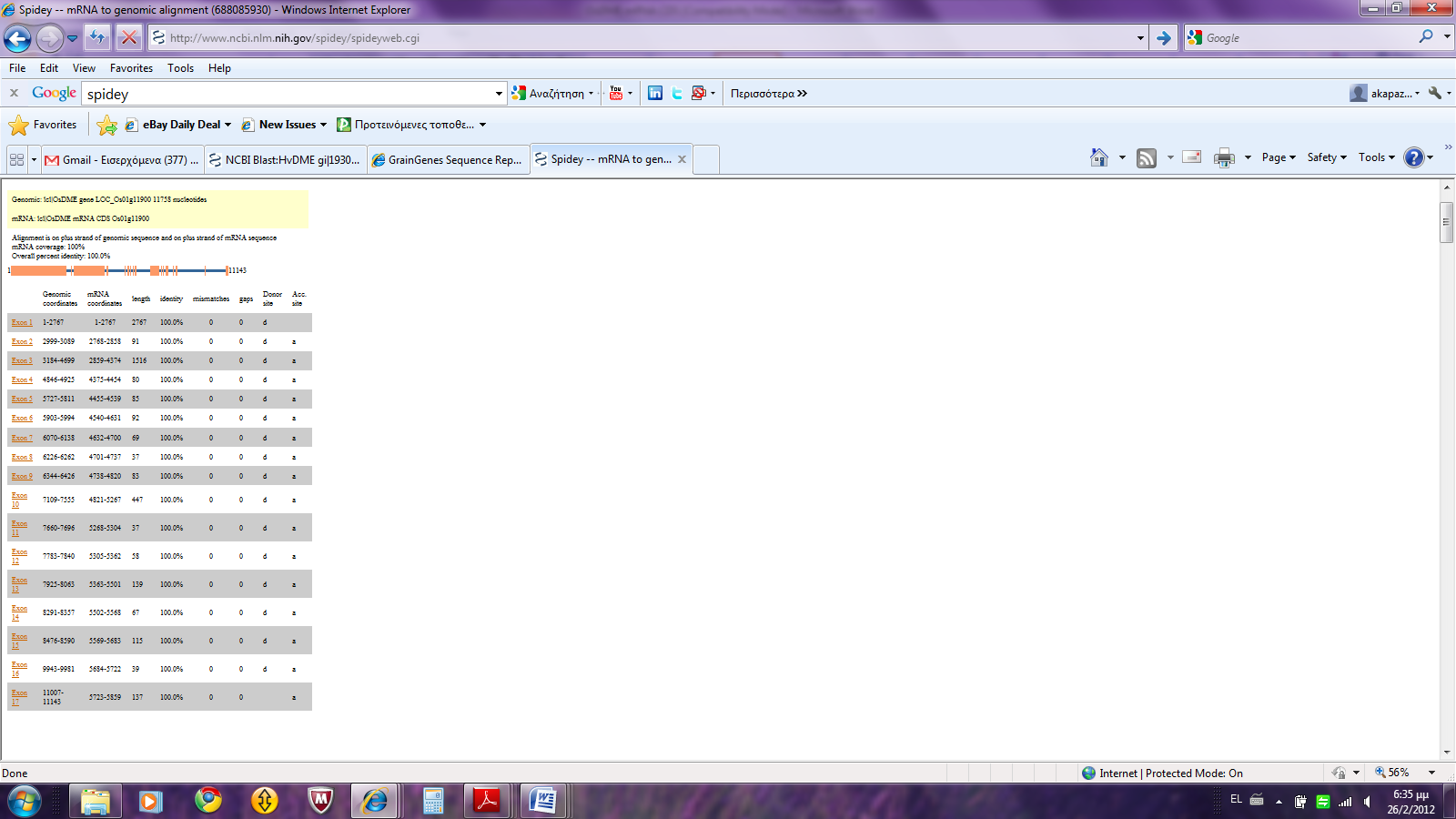


**OsDME**

**ZmDME**


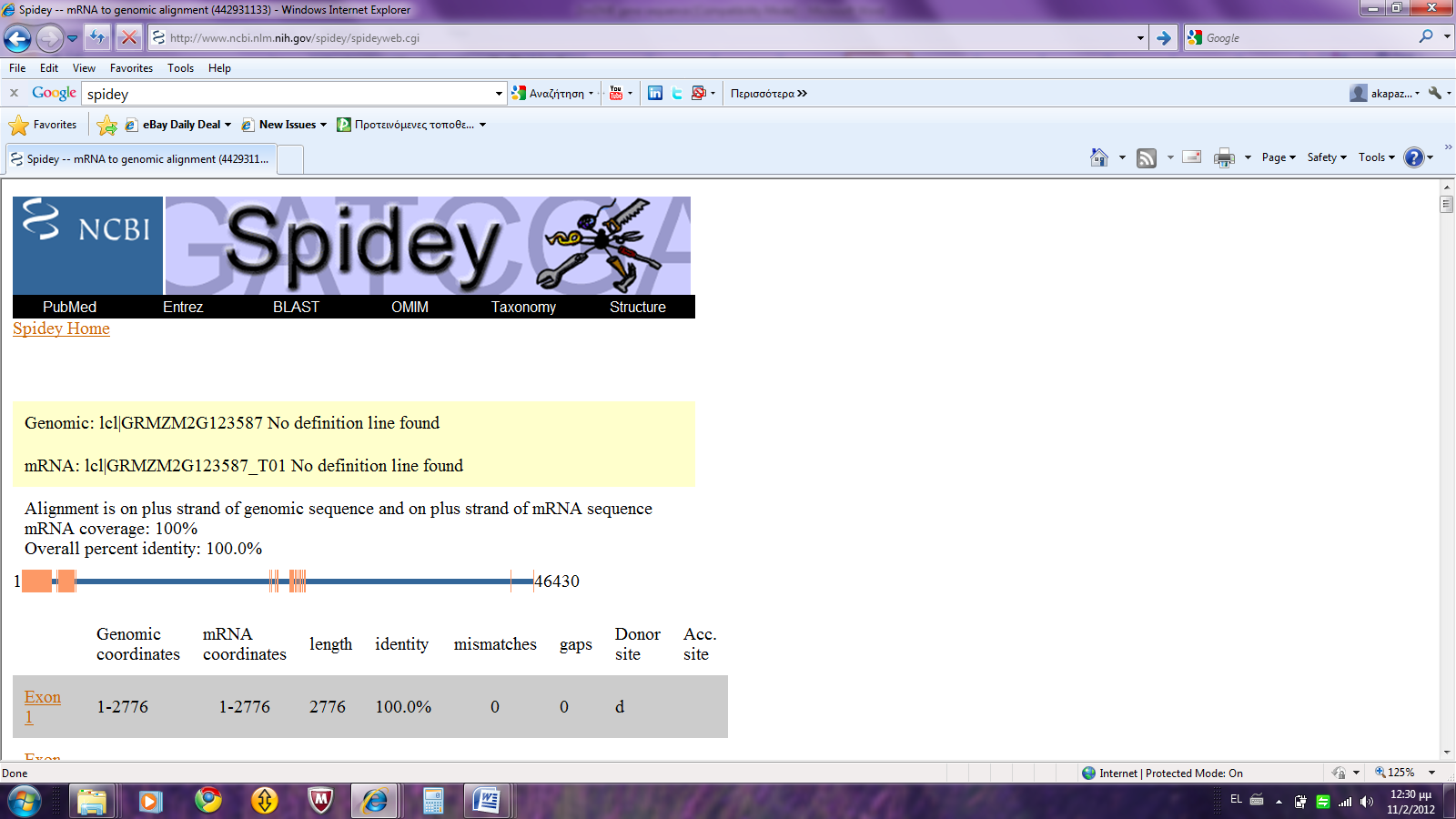

Supplement: Additional file 4 — Genomic organization of cereal DME genes. [file 1471-2229-13-172-S4.doc]
