# Supplementary material for: The study of a barley epigenetic regulator, HvDME, in seed development and under drought
Source: BMC Plant Biol. 2013 Oct 31;13:172. doi: 10.1186/1471-2229-13-172 (PMC4228467; doi:10.1186/1471-2229-13-172)
Supplement: Additional file 5 — The sequence of clone BAC 273i4, containing the HvDME gene. ATG and TAG translational start and stop codons, respectively, are shaded in pink. 5’ and 3’ LTR regions of the Copia sirevirus retrotransposon PTAES_CS_cons_maximus are shaded in grey. [file 1471-2229-13-172-S5.doc]

**Additional File 5**

The sequence of clone BAC 273i4, containing the *HvDME* gene. ATG and TAG translational start and stop codons, respectively, are shaded in pink. 5’ and 3’ LTR regions of the *Copia* sirevirus retrotransposon *PTAES_CS_cons_maximus* are shaded in grey.

>gi|193082831|emb|FM164415.1| Hordeum vulgare dme gene for putative transcriptional activator DEMETER, clone BAC 273i4, exons 1-17

GAATTCAGAATATACATCCTTCATTAATTTATTTTGAACTTACATAAACATAACACATACACATTGTATT

AAAAAACATTTCCTCCAATATAGGGAAATGAGNAGATAAGCTCATAAGCACAATATCATGTATAACTCAT

TCCATACAATGCATACACTAAACAAACACGAAAACACCACAAAATCGGGGATGAGATGACATATAGGTAG

ACCCCTACCTACGGCACCGGAACATAGGGCGATATTGTCACATGATTGACGGTGATTTTGCCGCTCCTCT

CTACACCCATCACACTAGGCACATGAAGCAACCCTAGATAGCGGTGACCGTCATGCCACCTGGAGGCTTA

GTAAATGCGAAATGGAATGTGGTAGGAGGAAAGAGATGGGAACATCAACGGGAATGAGGGGGGAGGGATA

AAGGGGGAAAAGAGGGTGGGGTGGGGGTGAAGGGCTATGGTAATATTAGGGGGAGGAGTATCGGCGATTT

GACAATAATAAAACATGAAAGTGTAATATCCAAATGTTGTATTCAAACTAAAATTTAGGAAGTTCAAATG

AAATCAAGACGCAAGATTTTACAGATATGCATTATCTGTACCAGCATAAATATACAAAAACTAGCAATCT

TTTGATACAGGACAAAAAAAGTAGGCTTTCCCACGGAATGGATTTTTTGCTAACCAAATAAAACTTATGA

CACAACGTTTGTCTAGTTTTATTGTCACAACAATCTCTACTGATAACAACTGATAAATAAAACCATTAAA

AATTTAAACACACATCATATGATGTGGTTGAACTAAGTTGAAATGATACACCATAAGCTTTGCATCCTCG

TTTCAATGATCGAGTAATTGACAATTGAGTCATCGATGGAGGACCCCATATGACAAGGTAGGGCAGCCAC

CCTACCTTGAATTTTATCTAAAAATGTATATACATAATGCATCAAACTACATTAGCAGCTGGTTCTGTAC

ATCAATGCATACTTACATAGTTTTTTCTTAATACGTGTACTTGCATAGCTCGGAAAAAATAAAAGGATAT

TCTCGAAGCACTGACCGTCAAATAACATTTTAGCTACGGTATTCGAGCCTTTACCCGTACGTTGGGTGCT

AGCCATGACTTCTAGCGAAGCAATTCGAGTGAATGCATGCGAAAAGAAACGAAGGATAGAGTCCACCAGC

CAGACACCGCACATCCTCACAAACCCTAAAACATACCATCAGTGCCGACGGATAACAATCAAAGTGCGAT

GGTGGCACACACTCACACACTATCTAGGCCAGGTTGCAGGGATACTGATGTGGGGCGGCACAACACCGTT

CTCTAAGGGGGGGTGCATTGTCGGCAAATATATAAGGTAGGTGACGGACTGGAAGGAGAAGTGGTTAGCA

AAGACAAAAGAAAAGGTACGAGTTTTAAAGTTTCTACATGTGTGCATATTGTGAATTGTGACGTTGATTG

AGTTTAGACAATATTGTCAAGTAGCTTGACAATCTAGTGATTTTAGTGGTATTTTCATGTTTTAATTGCA

CACTTGATAACATATTTTACGAGTTATTCGGAATTTTCATGTTATGGATTGTATCTCGTGATTCTATTTA

GTATCATTTGAAAAGACATGTATGAACTAGTTTGGCTTCCTATCATACTTCTTTTTTACCTTAAAATTCG

CCTGACCTTAATTTTCTTCGTCCTATGCCATGCCCCTTAATGGGGTTCATCAGTATGACGGTCAAATAAT

AATATTTAAATATTATGTTTGAAAAGGACACATGTGGACAACAACTAATAAAATTGATAGCTTTAGAGAT

ATATGATTAGGTGTGCCTAAGGCCCTTTCCAGTGCTCCATCATGGAAGGGTGCTAAACATGCCACATAAG

CGAAAAAATGATATGGCATAACAATTAAAGAGGAGAGAGGGCACTTTGGTGACCCTAGGAAGAACCGATG

CCTAACCATGAACCTAGGCAAACCACTTAAATGAGAGAAGTTGGTCAATGCATGCAAGATTTTAGGGGCT

AAATCATTAAATAAAGTAAGCTTAGCTACAACAAGTTAAGTAACTATGCATCGGGAAGTTGAGTTGCTAA

GCTATTTAATGCAATTAGATCTCATCTAAACATCTTTGCATTGGAAGAGGCCTAAAGTAACATGATAAAT

AGCTTGCGCCACTTTTGTCCACACAATCTCAAAAGTCACTCATCGCTATGGAAAACATTAAATTCAAAAA

CAATATAACTCACCCACTTAGGAGCTCTCACAATGACATGAGCTCCTCATCCACTAGATCTCACTCACCC

GTGGCCTCGATAGCTCAATTGTCTTACCACACGCTCTAGGAAAAATTTCAAGCGCCCACCCCACGCATCA

CATAATCGGCACAACATCTTCGGGTGGCTGCTCTAGAGACCGACCGAGATGGCCTTTCATTAAATGACAC

ATGATTTTTATGCAGGCCCTTGTTATAGACCTGGGAGGACGACACGTCGCTATGCGATTACTCTTGCAGA

GGCCACGACAACTTGTGGTGCTATTCCTTACTCTTTCCTTGATGTGCGTATATGGTCACGAGCCTCTTCT

CATTCATCGAAATATACCCTCGCTACCGACGGTAATCACTACTACTTATCTCCACCTACAGGGTTCACCC

CCTCTCCTCCGGCGTCCATCGCCACTCTGATCTGGGGCTTCCTCATGAACATAGGAAGAAGACGACCTGG

TGGTGGCCGCGCGACCCATATGGTACGATAGAGCTAGATCCATTGTTGAAGAACTACAATTATAGCTAAA

GAAGCAATATCCAGTTTCTTATTTAAAGATGCACAAGATCCTTTTTCTTCATGGTCAAGTGCCAGTCTCA

TTTTCACAACATTCTTTACCTTTCTCACAACCTAACACCGCACATACACAAAGCAAGTATAAAAAATACC

AAAAGCTCATGTATGAACCAGAACAAGCCAATCTATCACATGACCGATGGTGTTTTTGCCACTGCTTACC

ATCGTTCACCAAATTGTGCACATACATGCAACACTAGACATCAGTGGCAATTGATTCCCCTTGGTACCCT

AAGTGAATAAGCAATAAAACGTGGAAGGAGGAAGAGAGGGGATCGACAACAAGAAGGGGAGAGATAAAGA

GAAAGGGACAAAGGGACAAGATAAATAAGTGGAAGGGGCGAATGGTGATGTGGAGACCAAGAGGGAAGGG

GAAGTATAACTGATTTGATAAAAAAATATTGAAGTGGATGATCCAAATATTGTATTTAAATTGAAGTTAG

ACAAGTACAAATGAAATCAATTCGTAAAATTATAACTACATTCATTTTTTTATATAATGGTATAAATATG

TGTGTGCATATGTGTGTGTGTGTATGAGAAACCAATATTTTGATACAAGATAAAAAAAGAGCATGCCTCT

AAAATGAAAACCTTGCTAATAAAACGAGACATATTGCATGTTGTTTGTGTAGTTGCATTGTCACATCAAC

TCATATCGACGAGAACCGACAAATAAAATGACAAATAAAATGATTAAAAATCCAAATGCATATCATATGT

TGTGATTAATAAAAGTTGAACTCATTTGGAATAATCTAGAAGCTTTATCATCTCACTTCAACGGTCAAGT

AATCAATAACGAAGTCACCTTGAGAAGTGTGAAAATTAAATTTTAAATTTATGTTTGGTCGCCCGTTGAG

AGGACAAAAGTAACATTTAGATATTATATTTGAAAGGAACGTGTCTCTGCAGTAACTAACACATTTGACC

TGGTAGCTAGAGCGCGTGATTATTAATACACTTGACCTGTTAGCTCAAAAGGGATATCCGATCAGATGTG

GATAGATAATCTCAAAAGTAGTGAACTTCTATGGGGAAACACAAACTTGCTAAAGAAACACATATGTGTT

CCTTACTTAAAGACGTATGAGATCCTCTTCCTCATGGTTAATCATCGTCGTCATCCTCACAACACCATTC

TCTTTCTCATAACCTAACACCATGCATACACAAATTAAGCACAACAAAACACAAAAAATTGGACAACCAC

CCGACCACATCATATGTCGATGTCTCCACTTGATTGATGATGATTTTGCCACTGCTAACCACCATTGACC

GCATTGATCTCATAAATGCAACCATACACAACTCTAGTGGTAGATGCTCCTTGGCACCCCAAGTGAATAA

GCGATGAAGTGTAGGAGGATGAAGGAGAGGGGAACACCAACAAAAAATGAAAAGGTCAAGAGAGGAGGGA

CAAAGGATATGACAAAGAGTTGGAGGGGGAGCAAAGGGTGATGTGTAGACATGGGATTGGGGGGGGGGAG

TATCGATGATTCATTAAGAATGAAATATTAAAATGGATGATCCAATGATGTATTTAAATTGAATTTACAC

AAGTACATAGAAAATCAAATTATAAGATTGTATAAAAAACAATGATCAATCTTCAAATGTAAGATAACAA

AGAATGCCGGGTTATATAATAAAAAAATTGTTAACAAAGGAACCATTTCACCGTTGGTCTAGCTTCGTCG

TCACATCAACCTCTACTAAGTAGAATCAATACAAAACACTAAAAATTTAAACACCATATCATATGACATT

CTTAATAAAATTCAAACTCATTTGGTACGATAAAAACGAAAGCTTTAACACCTAGTTTTCAACAGTCGAA

TAATCACTAATGAAGTCGACCGTTAAGACGCGCGAAAAAATAATATTAAAATATTGCCTTTGAAAAGTAC

GCGTGTGCACGGAAACTAATACATCCGCCGATAGCGACACCTGATCATGTGTGGCTAAAGTAGGATGTTA

AATGACTTTCACCATTTCCGGGTACGAAATCTCAAAAGTAACTCACTGCTACGGAAACACAGTCTTGCTG

AAGAAGCACATGCGAGGTTCCTTATTTAAAAGCCGCAGCGGATCCTCTGTCCTCGTGGTCAATCACGCGG

TGTCATCCTCACAACAATCTTCCCCCTTCACTGCTCTTCTCTCTCTCTCTCACAACCTAACACCATTTGT

CCCCATTTCCCCAAAAACGAACAATCCAACACTCGACCGAGTCAGTGCTGCCTTCCCCCACTTGATTCGC

TCGATTCTTCGCCCCGAAGACCCTCCCCTCACTCGATCCTACTCACCCCCTCTCCCCGCGCGAGCTCCCG

ATCCGCCTCGCCGGCGGGCCGGCCGTCCTCGCCGCCGCCGCCGCCGCCGCCGCCGCCTCCGCTGCCGACC

TCGCTCGCTCGCGCCGCCCGTTCTCTGTGCTGAGGTCGTGGTTGCGCGGACGCAGCTAGGGTTGGAGGGG

TCGTCACCCGGCGTGGCACCGGCGCCTCCCCCTCTCCCCTGGAGCTGCAAGTGGGAGGTATCGTCTCGGG

CTTCCCAGATCTGACTTTGAGCGTTCCCCCCCAGGATTCTAGTACAAATCTGTGATTAATCAAGTGGAGG

GCCTGTTTGTTTGTTAGGTGTTGGAATATCGATTTTGTTGGATCGAAACTTGTGGTTGATGGAATTAGAA

CTCGAGCTGAAAGTTTAACGTTGGTCAACCTGATTTGTAGCTCAGCATGTCAAAGTTTGTTTTTGATTGA

TCGCGTTTTATAGTTCACAGAGTTGAAATGTTGAATTTTCGGAGAGGACCATGGTCACAAACTGCAACGA

CAGAGTTCCACAGGGGGCGGTTGGGTGTGATGAGCGATCACGCAACTGTACCTTTTTGGCTTGTTTAGAT

GGATTTGGAAAATGTTGCTTACATGGGTGTAAAGTGACTAGTGATTTAGCCATCCACGGGCAGTGGTGGG

GGCACGTTAGGGCCAATGTTTTTTCTTGCGCCAGGATAGTTTATGTACGACAATGCTTTCGCAGGAAAGG

ATAAATGCGGATATTTGAGATAGTATGTGTGAAGGAACTTCACAGAAATGCTTATGATTACTCCAGTGCA

TAATTTATTTTGTTCTTTTTCATTTTGTTGTTTTGATACAAGTGTCTAAGGAGTTTCCATGCTGGCCACT

GATAAAGTGATAATTGGCTCCTTGCTTTCAGTAATAGTGATATATTTTAGTTAAATACAATGTCTTGTTA

GTGTATATATGACAGTTGCTTCACTGGCTGTTTGTAATGGAGGTTGATGCATCGTGCCTTGATATTTTGC

AGCTGGAGAGTGGGAGACCAGATGGGGGGCCCTGCCTGATCAAATCCTGCTTTTCCTCACGAGTGACAAG

AAAGTACGCAGCAACACATGGCACACGGGCTATTTGTGTATGCCAGGGAGATTAAGTAGACTGAGGAAAT

ATGCAGGGTTTTGGACAATGGCTTCCTCAATCGCAGAGTGCAGCCGATCTTTATTTCTCAAGTATTATGT

CATCTCAGTTGGACACTTCCATAGAGATGCAGACTAGAAATACTGAAGTTGCAGTGTTGGAGAATGAGTC

TGCTCATTCGTTCGGCATTACAAATGCTGCTGGGCCAATTGAAGTCACCAGTAATGATGCTGGGACAATT

ATAGACAATGAAAATGTTGCTGAACCGACTGGAGGTATTGACTTGAACAAGACCCCACCACCGAAGGCTA

AGAGGAAAAAGCACAGGCCAAAGGTCTTGAAACCATCAAAGCCTCCTAAGCCTGCAACTCCAAAGCCCTC

CAAGGCAAAGGAAGAAAAACCATCTGGTAAAAGAAAGTATGTCCGTAAGAATACGCCAGCAGGCCAACCT

CCCCCAGAACAGATTGCTGGGTCACATTGCAGAGCTAACCTGAAACCAGCTAAGCGGTCTTTGAATTTTG

AGGGGGAAGTTCCACAAGAGAACACACATCCTGGATCCCAAGCTCAAGTGGTATCTTGTGATCCCAAGGA

GTATCAATCATCCATGCCTTCTACCGGTCAAAGAAATGTCCAAAGTCAGTTGACATGCCATTTGGATTTT

ACCACCAGTTCAATGTACAGTTCAGCCAATCAGATGGCTGATACACAGCTATTGCCTGCAGATAATATGA

AAACATCAATTTACAATTCAGCCAATCAGATGGCTAATGCACAGTTTTTGCCTGCACATAATATGCCAAA

AGGAGTATTATTTGACCTCAATAGTTCGACGAATCAGATACAAAATGAGTATGCCAATTTTTTGGATGGG

TCTGCGCAATTTTTTCAGTCTGGAATAACAGAAACATTACAAACAAATCCTTTGCTAGAGCTCTGTGCTG

ACATGCCAGATAGAAATTTACCTGATCTCAACAGTTCAATCACTCTAATGCAGGGCATGCCAACTAATTT

TACCGAATACTTGCTTTCATCGTCCCAAGCTTCTGTAAGAGAAACACATATGGGCAAACAGATGCCTAAT

TGTCAAAGAATGCCAGAAAATCCAGTTACATCTGCTCAGTGTTTTGAAGGGGTTGCAACGAGGGAAAACT

TCAATCTTAATTCTTGTTTAAGAGAAGGAGGGGCAACCAACCAAATGTGTCATGGCTACAGATCGACACA

AAACCCAATCCCACCTCCCAATCAAATTGAAGGGCATTCTGCAATGGAAAATTTGAATGGGCTTGGAAGG

ATAAGTGACTACTTAAAGTTTACCAGCAGTCCTAGTCCTTACAGGCAAACAGGTGGTGCACTTGGACTTC

ATGGTTCACGCAGCTCGTCACATATGCACGCATTGGACACCAGAGAACACAATGCTTCCAACAGTGCGTA

TATCCCATTAGGTATGAATGGTGACCAGCAGAGGAATGGATGGGCATCTGTAGATGCGTGCCATGCTGCA

CCCTCTCAGGGGTCATATTTTCCTGAAACCTACAAAAGACTGAGAACAGATAATTATAGTAAATACCTGA

ATGGAGCTGTGGGCAACACATCCACCCCATCTATGTATTTGTCGAACAATCAGAATACAAATGTGGTTTC

AGCTATCAACTCTAATGTGTTTACCCTAGCTGATGCTCAAAGACTGATAGCCCGTGAGAAATCACGAGCT

TCCCGAGGAATGATTAATTTTGGAGGAACAGGATACAATACGGTTAAAAGACCGGAAATGATCGAAGAAC

ATTATATACCTGCTATCCATGGCACTGCATGCAGTGATTCTGTTGAAGCACCTGTTAAACATTTTAGACA

CATAACAGAAAAAATTACGCAGGTGCCTAGTAATCCAAACACCCTGCAAAGTCAAAACTATAGTCCGAGA

ATTGGAAGTCATCAACAACAGTTTTGGGAAGGCAACACAATTGAAGTGTCAGATTTGCCTGTTGAACAGC

ATAATCAGAGCACTGCCCCTCAGGATGATACTCGGAATAGTTTTTGTATTGGTCCTTCTGATCAGCTTGG

CGGAAGCATTAATGGTGACATTTCTAGGCTCCCTGTCACTCCAACAGGCCAATCAACAGGTAATGACACT

CTGAAAAAGTTTGGTTCCCAACTGGAGACTTCTGGAGAAGTTATTATGCCCCTTACCAGTCCAAGAAATT

CATCACCACGTACTGATGTTCTGAGAAATGAGAATCATCAAGTGGAGGTTTGTGGAGAAACTACTGTGGC

TAAACCCAGTGAGAAGCGAAAAGCAGGTCGACCCAGAAAAGAGATAAAACCTGGTGAGAATCCAAAGCCT

AGAGGTCGTCCAAGGAAGGAAAAGGTGGTTGGTGCAGAACTTGCATCCAAAGGTAGCCATACTGATCCAT

TGCCAAATGTGGATATCTCTGTTATCTCCGGACCTCATGCAGGGGAGTCTCCTGGTCCCAAAGGGATTAA

TACGGAACGAAGTGGAGAAAGCTTTCCTGGATCTATAGCACCTCCAGTGGATCCTTTGGATCTCATAATT

CAAAAGATAAAAGTGTTAGACATAAACAAATCAGATGGCATTGGGTCAGCTGAGCCGCATGGTGCTCTTG

TCCCTTACAAAGGAGAATTTGGTGCGATTATTCCATATGAGGGGAAAGGGAAAAGAAAATATGCTCGGGC

CAAAGTGAACCTTGATCCAGTAACTGCTCTAATGTGGAAGTTATTAATGGAACCAGATATGGTTGATGGT

TCTGAAGGAATGGATAAGGACAAAGAGAAATGGCTTGATGAAGAAAGGAAAATATTCCGAGGGCGTATTG

ATTCATTCATTGCTCGCATGCATCTAGTTCAAGGTAATGCTACTATTTCAAATCTTCATTCCTGACAGGG

CCTTCTTTATGCTACTCCACATATTGGATTCCAGAACCTTATAGGGTCTCATGTTCGAACTCCTGACTTC

GCGCTAATGGGATGATCACAGATCATTAATGCTTTGGGTTGCACTGTGTTGGATGCGATGATGGCTTATA

GGTTTTGCAAAAAGGTACCTAACTCTTGAATGACCTCTGTCTGTTTGCAGGAGATCGTCGTTTCTCTCCC

TGGAAAGGATCAGTTGTGGATTCAGTAGTGGGTGTTTTTCTCACCCAGAATGTTTCAGACCATCTTTCCA

GGTGAAATAAAGCTTGCAGCCTATTTGAACTGGAAGCTCTTTTTGACTAGCATTCCGAGATTATGTTGTT

TGTCTGACTGTTGTCTTCTACTGTTTTGTAGCTCTGCGTTTATGGCCCTTGCTGCAAAATTTCCTGCAAA

GCCAGAAGTCTCTAAAATATCTGCAGATAGGATGTTTCACACAGCATCAGAGAACGTTGGTTGTTCTGGA

TTGTTTGGCGATTCTGTCAAATTGCCGGGCGGTATTCTTGTTGAAGAGGCAAGTAACACAACAGGCTCCT

TAGTTACAACAGAAGAAAAGGAAGGAAGCAACAGTTCTGGATTGTTTGGAAATTCTCCTGGAGATGGAGT

AGACTGTACAGCAGGGGTTTATTGTAATTCTTATGGGACGCTGCCGGTTAGGCTGCATGAGGGCAAGACA

CCGGCCGTGGGGACTGAAAGTGTTGTTGAAGTCGAAGATGGGGCACTGGAGGACGTTGTTTCATCACAGA

ACTCTGCTATTTCATCTCAAAGTTCCCCTGATTATCTGTTTCACATGACCGATCATATGTTCCCAAGCAC

ATTGCTAAATTTTACAGCAGAAGACTTTGTTGGCAGAAATATGGCCAATGGTACAAGCAATTCAACTACA

TATACAGAACTTCTAAAGATGCAAGAGCTAAAGAGCAAGCCTAGTGAAAAGGAATATGATGGAGTTCCAA

TACAGTGTACAAACAGGGGTTCAATCCCCAGTGAAGTACATAACCTCAGCAGCAAGGCTCAACCTCTTCA

TGCTTCTGGCTCTTATCACCAGAATGTCCGAGCTCATCTCCCAGATATGACATTTGGCAGTGACTTAGAG

CGCTCAGTATACACTGGCCTCAATAGAACAGATGATTCCAGGGTCTCACCAGCTGAGATCAGATATGATT

GTTCTTTATCTTCTCCCGGAATTGACAGTGAAAATAAAGCTCAAACGACTGATTCTTTGACTGCCCTTTT

ATATGGTATAGATGGATCCTTAAGTCAGGACAAAATTCCTTACCCTTCCATGGCGACACGAGGAGCTGAC

TCAATTTCAATATTAATGGATAAATATTTTTATCCATCAAGTTCAGAGACGGTGTCATTTGGTAGGGAGC

AATTGTCCTGTGAAAATAATCTTCAAAGAAATGACGTTGTAGCTGCATTTGCGAAACAGCATGAAACATT

GAATCTGCAAGAGGAATGTACTGCCAGAGCAAAGCAAATTGGAGGTGAAAATTATCAGTCAGGATGTAGC

CAACAGTATGGCAATGTTGGACTTTCATCAAACATGGATGGGAGTCATTGTTCCTCAAACTTATATCAAA

ATGAAAAGGCAAATTCTGAACTTCTACAGAGGGTTGCTTCAGACTCAATAGAGAAACCCAAAGATACTAA

TAAGGCTTTACCTGAAGTTCCTGCTGACAGATCAAAAACAAAGAAGACAAGGGCTGGTAAAAAGAGAACA

TATGATTGGGATATTTTGAGAAAAGAAGTTCTTGCCAATCGAGGAAATGAAGAAAGAAGTGAGAATGCAA

AGGATGCACTTGATTGGGAAACAATAAGGCAAATAAATGTGAAGGAAATATCTAACACAATTAGAGAGCG

AGGAATGAACAACATGCTTGCAGAGCGGATAAAAGTAAGTATGCCAAAAAGTAGTTATTAAATGAAGTAC

ACTATAACCCTAGTCATATGGACATGTGGACAATCCAATCTAGATTTGCCTATTTGTTGAAATTGTCATG

GTTCGGTCATGTATACTCATCTTTAGCAATTTCCATTGTAGGATTTTCTAAACCGGGTGGTGAGAGACCA

TGGGAGCATTGACCTTGAATGGTTGCGACATGTCGATCCAGACAAAGCAAAGTAAGCTACTCCTTCCGAT

CCATATTACTTGTCGCAGCTTTAGTACAACTTTGTACTAAAGTTGAGTAAAGCTGTGACAGTACAATGCT

GTACTAAAACTGCGACAAGTAATATATATATTGAGCTAACATTTATAACTGTATTTGTTTGTTCTATACA

TTGTCTTCCATCATTATTATTTGAGGCAAAATTATGCAGGGTTCTCTGTTGATTTTGTAACCGGGTTTCG

TTGCTAAGATTTGTACCTGTACTGGTTTGTGTTATTGTTGTGAACCATCATCCACAACGGGATAACTACT

GTTTTGAAGATAACCAGGCAACATGGCTGGCTGCTTGGGATTGAGTAGACATAGGTTGAGATGAGAGAAT

AACTTTGAGATAATAAATTGAGTCTTCTAGCTGGTTTCCTCGAAAGTTACTCATCCAACAGTTCATGCTG

CTAAGGATTGGGTTAACATAGGTTGAAAGAGAGAACAAGTTGGAGATAATAGATTGATTTTTCTTGCTTG

ATTCCCTCAAAAGTTACACAAGCCAACACTAAATAGTACTCCTACCCCGTAAGTCTTAATTTGACCTGTT

GCTATATTAGGAAACCTCTTAGTAGATGTATACAATCCATGTTTCTTAATCCTCCTGAACTCTTCGTAAG

CATTCTATCCTAATTCTGTTTCTTACACACTTTCTGAATTCAAGTTGTCTTAACACCAAAACATCCTTGT

CTTCTGTGTCTTGCTGGCCACTCAATGCATCTAATGCTGCTTTGATGCAACATGCTAATGTCCACATCAG

TTATTTATTTCACTACCATTTTGCCACATGGTTCTGTTCAGCCAGACTAATTTGACTTGTTTTTGCTGAG

GATCCTGATTGTACAAAAACAATTAAAAAAACATAGACAACCTTGTTGAGGAGGTGATTTAACAAAATCC

CACCTACAGGAGTCTGATTAAATTGTACTCCTTCTGTAAAGAAATATAAGAGCGTCTAGATCACTAAAGT

AGTTATCTAAACACTCTTATATTTCTTTACGGAGGGAGTAGTTAACCTGTCAACTCAGCTGTGACTCTTA

AAAGGGCCTCCATGACAATTATGTATGAATGGCTTTAACTGCACATAATCTGGACTGAGGTACTTGATTG

ATCATATCAGAACAATGTAGGGTCTCTCGTCTAGATCTGTAATTTTGCCTTACACTCAAATTTTATTATA

CTTAGCCACATATACTATTACAAATATGTAGACCTATCACAAGAAAAATAATGGTCACATTCTTATGAAA

AATAGCTGTATAGCCTTTAATTAACATACCCTGTTACACATTAATGGCCGAAGTTGCATTTTGGGACTGC

AAAGTAAAGACTAGTCATTTTTGTTTTGGGCTCGAAGTAGTATCTGGTGCTCCTTTTCTTCTTTGAACAT

ATACAAGATGCTGAACAAGAACTCAATGAGAAGTGGAACTAATAGTTCACTGACTGCTTTCTTGTGTAGT

GTTTCCATTCTTGTTACATATGAACGCATATAAAAAGTCCAATTCAACACTGCTATAAGTCATAACTGAG

TGCATAGACGCATACATGTTCGCTATTCAAAAATTTATCATTGATGGACTCATAGGGTCTTGTGATGATG

GGAGCCCTCAAAATGGTTTTTCAACATGTAAAAGGGTTATTCTAGTAGTTATGGACCATGTGATCTCGAC

CTTAGATGAATGTTTTTTGTCCCATTCTCTTGTTTGACCGTTCATGTGGGAAGTTTGCATGGTCGACTGA

AATAAATGTTTGCACCATATTATATTATGTGGCATCGCGCAATGGGCTAAAATGCATATAAATTTCTTTT

GTTGAACATTTTTGGCATTTAAAAATTATAACCTTGTTGTTGTGTCTCTTTCAGGGAGTACCTCTTAAGC

ATTAGAGGTCTTGGACTGAAAAGTGTAGAGTGTGTGCGTCTTTTGACACTCCATCATATGGCTTTCCCTG

TATGTCCTTCCATTCCATACTTTGTTAAATATTATTTATTTAATTTTGGAGGTTTATCAGTATCAGTTGA

TTATGTAGGTGGACACAAACGTTGGTAGGATCTGTGTGAGGCTGGGATGGGTCCCACTTCAACCCCTACC

CGAGTCTCTTCAGTTGCATCTGTTGGAGCTGTAAGTGCCCCAATCCTTGTGGTTGGTTTGACTAGTATAT

ACACAAATTACTGAGTTTGATCATGCAAATTTGAAGGTATCCGATGCTGGAGAACATACAGAAATACCTC

TGGCCTCGATTATGCAAGCTTGATCAACGGACATTGTAAGTCTTAGAAACTTAATTAATTACTTATTTTG

GAAAAGGTCTTTATCTGCAGGTATATAGTTACAATTTACTATGTGCAGGTACGAGCTTCACTATCAAATG

ATAACGTTTGGAAAGGTAAACATGATATTTGCGAGTGCATTTGCTGACTTGCTGTGTGTTATTGAATTAT

TTTACTCTGTTTTCCCTGTTAGGTATTTTGTACAAAAAGTAAGCCAAATTGCAATGCATGCCCAATGAGA

GCTGAATGTAAGCACTTTGCGAGTGCATTTGCAAGGTAATTTATCAAGATATGTGTACATTTTGATATAC

ACTGTACCGGGCTAATCAACTATGCATCTTGCAGTCTGGTATTTAAACTTCTATCATGTTTGTGTGACCT

ATTTTCCTGTGCAACTGTGTATTGCATTGTGTTAATTGGTAGCTAAAATACCTCCCTTGTGGATAAAAAG

CTAACCATCTACTTTCTTGACGGCAGCGACTGATAATATTTCCCTGCAAAAAAATGTTAGTTGGTTTCGT

TGGAAAAAATATTGGGTTGTTGGAATTTTTTTTTATTTCGGTCCAATAGATTTCACAAACTTAGCGAAAC

TATTAGTTCTTTCGATAGAACAAATAAAGTGCCAATGGATGGCTTGCTAAAAAGTTTGTTTCTATTCCAA

TTTCAATAGGAAAAGTTCATTGCTAATAAGTTTTTTTTTGATACAGTGCTAGACTTGCTCTTCCTGGACC

TGAAGAGAAGAGTTTGGTTACGTCAGGAAACCCCATTGCTTCAGGGAGCTGCCAGCAGCCATACATAAGT

CCTATGCGTTTAAATCAACTTGACTGGAATGCACATGCCCATGACCATATTCTGGACAATTGCCAGCCAA

TCATTGAGGAGCCAGCAAGTCCGGAACCAGAACCAGAGACTGCAGAGATGAGAGAGAGTGCCATAGAGGA

TATTTTTCTTGACGATCCTGAAGAAATTCCTACAATTAAGCTTAATTTTGAGGAGTTTGCACAGAATCTC

AAGAACTATATGCAAGTCAATAACATTGAAATGGAAGATGCTGATATGTCAAGTGCCTTAGTTGCCATAA

CTCCGGAAGCTGCATCTATCCCAACTCCTAGGCTCAAGAATGTTAGTCGCCTAAGAACAGAGCATCAAGT

GTATGTCCCTGTCTTCCACAAAACCAGTATTATTGATGATAACTATAGTACCCTCGTATCTTTTGTTATA

TTGCTAGTATAGCAAAGAAATTTACACAGGATCTGTATTGAAATGTTCTCATTATTTGGCTTCTCTGTTT

TCTACATGGCTGCAGCTATGAACTGCCGGACTCACATCCACTTCTGGAAGGAGTGAGTTCATCGGTCATT

ATCGAAATTTGTACTGTGCTAAATGTGGAATAAGATTATAATGAGAAATATCTTTATTGTGTGCAGTACG

ACCAAAGAGAGCCTGATGATCCTTGCCCGTATCTACTCTCTATATGGACCCCAGGTAAGAAGCACATTAT

AACCTGAAGTATTATGGCTTTCATACTCCTGCCCTCAACTGCTAATGTTCATTCGAATATAAAACAGGCG

AAACAGCTCAGTCAATTGATGCACCCAAGACAGCATGCAACTCCAATGAGAGCGGCAAACTATGTGACAG

CAGTGCCTGCTTCAGTTGCAACAGTATGCGAGAGGCGCAGGCTCAAACAGTCAGAGGAACAATTTTGGTA

CGCACCTCATTTGTGTAATATACTTGCCAGAACAGTACTAGTGGATGGCCAGAAAGATGCTCTGTAGGAA

ACCAGTGTCCAACTCCACCATTGATTAGGAAGACTCCATTACTACGATATATCAAGTTAAGAAGCTAATT

CGTAAGCTAGTAAGGAATTATTAGAGTTGGTGTCCAAAAAGAGATTAGCGCATCCTCCTATATGCATCGT

AACGGATGAATACTATTCTCTCTCATTCTCATCTCATTACGTCCATCATACCCCTTCTAAACACCAATTT

CCTTAAAGCCTGATGTCTGGCCTTCCTCTACATGATGGTTATCTTTAATGGGAACCCCAAGGTTCATATC

AGCTATAGATTTCGGGAAATGATTCCTAAGTTGTCTCTAAAATCCTGAACTCTTAATAGAAGATGTGTGT

TCTAATGGAGACAAAATGATTTGTGTTAAAAATCCTTCCTGTCTCAACTAATTGTGTGATGTATTAGCGG

GTATGCAATTTTGGTGTTTGTGCATGGCATGGTGAGATCGATTGGTCTGATCCTTTTGAGTATATGAGAA

GCAGAGAACGGAGCGTAGCTCAGTTGGCAAGGCGCGGGAGTTTGCAGCCAGCCCACCAGTGTTCGAGTCT

CGGCTCGAGCGCTTGGTGCTCACGGAGTTTTTTTCTGTAAAAAATGCCAACAGGCTAGTCTAGCCCTGGT

TGGTCTCGTATATGAGAAGCAAACCGATGACTTCCTCTCATTTCACGTATTGGGCAATTTCGAAGTCAAG

AAACAGTGGCATTACTTATATTGTGTTTGGTTGAGAACCGCTATGGCCCCCTGTTGTACATCAAAATTTT

GGATTTATGTATGTGTCGAACATGTTGTAGTAGCAAGCTACCTGGCCGTGAATTAATCGATGGAGTACAT

TATCTACTGTACAAAAGTTCAGTATGTCAAATATCGATGATCCAATGACCTTACATATTACTATCTTCGA

AATGGTAGTGCCTAACTATCTTTTTGTCGAATTAACTCAAATAGGTACCGTGTCGAACAGCTATGAGAGG

AAGCTTTCCACTTAATGGGACATATTTCCAAGTAAATGAGGTAATGACTGAGTTACATCTGTTCATAGTT

GGAATGGTATAGTAACATCAACAGAAATACAAATGTTCCTGATATTATAGTTACTAGCTGTTCAAATGTT

ATACTGCAGGTATTTGCTGATCATGACTCAAGTCGAAACCCAGTTGATGTTCCGAGGAGATGGATATGGG

ACCTCCCTAGGAGAACAGTTTACTTCGGAACTTCAGTTCCTTCAATCTTTAAAGGTACATCCCTAACGAG

AACCAAGGATTCAGCATTCATTTCCACTGTGGTGGGCTTCTTATCTTCCTAAACAAACCATGTAGATTGA

CTGAGAAGTTTGGGATAGGCAAAATAAAGTACTGACAGTATAGAACTCCAGTTGATGTTACTTGCTGACT

ACTCCCTCCGTTCCTAAGTATAAATTCATTCATTTCACTTCGTATGTAGTCCGTAGTGAAATCTTTAGAA

AGACTTATATTTAGGAAAGGAGGGAGTATTGACTTATATTTAGGAAAGGAGGGAGTTCATTCCCACTACT

TGTTTTGTGGGACTTCCTATAGTCATGGTAATATGGCATGAGATTAGTTTTTGGCATATATGAACATCTA

GCTGTCCCATGCAATACTAGTACCAACTGCCGTCAATCTTACTAAAATCTATGAAGGCACAGCACCAACC

TGTACTACAGGATTTTCCAACCACCTTGCCTGCGGCCACCGCTCCCTCCTATCCAGCCAACCACCCCCAC

CTGCAATTTTATACTCTTTGCTTGAAAACCGCTCAACAACAACAAAGCCCTTAGTCCCAAACAAGTTGGG

GTAGGCTGGAGGTGAAACCCAAAAGATCTCGCAACCAACTCATGGTTCTGGCACATGGATAGCAAGCTTC

CGTGCACCCCTGTCCATGGCTCGTTCTTTCGTGATACTTCAGTCCTTCAGATCTCTCTTTAGGGACTCCT

CCATGCCAAGTTCGATCTCCTCGACTTCTCTTGACATTATCAACACGCTTCAGTCGTCCGTTATGCACTG

GAGGCCTGCATTGAATATGCGCAAACCATCTCAGACGATGTTGGACAAGCTTCTCCTTAATCGGTGCTAC

TCCAACTCTATTTCATCTATTATCATTTCGGACTCGGTCCTTCCTTATTTGGCCACACATCCATCTCAAC

ATGCGCATCTCCGCGACACCTAACTGTTGAACATGTTGCCTTTAGTTGGCCAACACTCAGCGCCATACAA

CATTGTGAGTCGAATCGCCGTCCTATAGAACTTGCCTTTGAGCTTTTGTGGCACTCTCTTGTCACAGAGA

ATGCCAGAAGCTTGACGCCACTTCATCCATCCGGCTTTGGTTCGATGGTTCACATCTTCATCAATGTCTC

CATCCTTCCGCAACATCGACCCCAAAATATCGAAAGTTGTCCTTCTGAGGCACCACCTGCCCATCAAGGC

TAACCTACCCCTCCTCATCCTTGTGCCTAGTAGTACTAAAACCGCACCTCATGTACTCAGTTTTAGTTCT

ACAAGGTCTAAAACCTTCTGATTCCAAGGTTTGTCTCCATAGCGCTAACTTCCTACTGACCCCGTCCAAC

TATCATCGACTAGCATCACATCATCCACAAAGAGCATACACCAGGGGATATCTCCTTGTATATCCCTTGT

GACCTCATCCATCACCAAGGCAAAAAGATAAAGGCTCAAAGTTGACCCCTGGTGTAGTCCTATTTTAATC

GGGAAGTTATCAGCATCGCCATCGCTTGTTCGAACACTTGTCACAACATTATCGTACATGTCCTTGACGA

GGGTAATGTACTTTGCTGGGACTTTGTGTTTCTCCAAGGCCCACTGCATGACATTCCGCGGTATCTTATC

TTTGGCCTTCTCCAAGTCAGTGAACAACGCTACTCAAATATTATATATTTCAGTATTAGGGCTGTCAATG

AGCTGAGCTCGAACAAGCCAATGTTAGTGCACAAGTTGAAGGGACATGAAGAACACAAGATGGAAGAGCA

TGTACACTAATCGCATTATCAAGATTCAAAAACAGAAGAAATAGAGGCATGGTATTGAATGGGGTTCAGG

CTATCAAATCACTGGACATGGTGTAGGTCTCAAAGTCTTTGCTCATGGTACTGAAGGGTGTGCAGTCATG

TAGAAACTAGAAACTCATTGTGAAAATCCCATTTGTGCATCTTTGATGAGGTTTTATTCCCAAGCAAAGC

TCATTTTCATTTCCACCGTCTAAATAGACACATAGAAATATGTTCGCATGCATGATTAGCACATGTGTGG

AGACATTAGGTGGCGATGTTATGTTGGGGCTTACGTTGATGTTGGAGTTTCAACTACAAAATGGAACGAT

GAATTGAAATACTATTTATATGAATGAAGAAATCACTTAGCAGTTGTAATAAAATTTGGTAAACCTTAAC

TTCTGCTAGAAATTGGCTAATGTGCAAAAATGTTTAACTGGGAAGTACTAGGCGTTATGGAAAATGGTAT

CTTCAGCAAAATCCTCACTTCCAATTTTTACCTGGTTTTATGTTCATGCATGGGAGATTGCATCTGTTTT

AAGCACCGGAATGTACTCCAACATCCATTTGTCCTGTGTCAACGTTGAGTTTTATACTAGCATTATTAGC

CTTGTATAAATAAACAATATTTTTTTGGCGGATTATCTTCCACTATACAAATATAAACAAATGCCAAGGG

CTAATAATAGGATTACCACATCGAGGATGTGTAGATGATTATACTTTGCATGTTTCTTTTGCAGGTTTAA

CAACTGAAGACATACAGCAGTGCTTCTGGAGAGGTAATCATCTAAATGTTTTTGTCATTTATAATAAGGG

GTGTGATCAAAACTATTTCCTGGAGTCAAATATTTCTTAACTTATTTTATGTTCCAGGATGTACCTCAGA

TGACTAATATTTGCCAAAAAACTTGTGAAGCCCATAAATGCATAACATCATGGAAGTATTTTTTAATACA

AAATTAACTTATGTAATTTTACACAGCAGATATGACTACTTCAAAATATATTGATCAAATATGAACTTTG

CTGCACGAGACAGAAACAGAAATTTATGGTGTGATTTAATAGTAACAGCTCCCAAGAGGATAAACATGTC

TTGGATGTTGGTTTTATCTGTCAAACAAAGAAAAACTATGGGAATTGGAGGATTATCATATCTAGAAATA

AACAAAAGTAAAATAAATAAATATCAATATGGATAGCGTATTTGTCCTTGAGGATTGTTGGAGGGGGCTA

TAATGGGGTAGAATGCTATTGATGAGATTTCATTTGGAAGATTTCCAGTAACATTTTTTACTCGGGAAAG

TAGATGTTTATATGTCTGAACGAACATCTGCAGGATTTGTTTGTGTGAGGGGCTTTGATAGGACATCAAG

AGCACCAAGACCACTGTACGCAAGGCTTCACTTTCCAGCAAGCAAAATTATCAGGAATAAAAAGGGCGCA

GCTTCTGTAGGCATAGATGATGCATAGCCCGTCAAGGAAATAAAAAAAAAACCCGAGTTACATAGAAGAC

CCTGCCAGAAGTTCACTGACATGAAAATGGATCACTGACCAATAAGTTGTTGTCGCCAATTCAACCATCT

TTTAGCCTTCCGTACCTGCGAACGTTATGAATGCCAGCCACTCGCGAATTTGTGAAGCTGGCGTTGTTCA

GCAACATGATCCATTACTACACAGATTTATGCTCATTTGGTTCCCTCCAAGAGTTCGATACAGCAAATGG

GGAGGGGAGAAAGTCTGCTGTCTGGGCCATTCTGTTTGTTGTTTTCATGGGATTTGAACCAACTGTTGTA

CATTGGAGACGTCATCCATTCAAAGTTGTTCATGTTGTTCGGTAGTTCATGTGAACGTTGTCAGATTATT

CGTCAATTATTTGATTGTTTCATTGATGAGGCCATTTTGTTCTCTTAACTCCTGTTTCATTGATGAGACA

TTTATATTCATTCGTATTACTCCCTCCATGTCAAAATATATGCATATTACTTCCTACATTCCTAAATATA

AGTCTTTTTAAAGATTCCCCTAAAGGACTATATATAGAGCAAAATGAATGCATCTATATTCTAAAGTATG

TCTATATACATCTGTATGTAGTCACTCAGTGGAACTTCTAAAAAGATTTATATTTAGGAATGGAGGGAGT

AGTTGTCTTATAAGTCAAACTTGGTAAATTTTGACCAAGTTTTTGTGAAAGGTTATCAACATTTGCAATA

CCAAATCTATCACTAGATTCATCAAGAAATTTCATACTTTTCTATAAACTTGCTCAAATCTAACTTAATT

TGACTTGGAGCAAATCTAATATACAATGTGTTTTTTGTAATGGGGGAGTATTATGCCAAAATTCGACTGT

CTAGTGCTCCTTCATTTTTTTAGGGCGCATTTGGTTGGCTGCATCGATTATGGGGTGTTTGCATAGCATT

TTCAGATGGAACTGGTTGAGAAAAAATAACATCAAAATCACCATGTATATTGATTGGTTGCTTGCATATA

GATTAGCTGCATAAGGGAACTAATTTTGACCCGTTGTTTGGTTGGCCGTATTGCATTAGGCATGCATAAA

ACATGTTTTTTGGTTGCAACCTGCATTAGGTGCTTTCACCACTTCTCATTAGTGGTGATCTTATCACACA

TACGACCTAGTATCACTCCTAGATATGAAACGAATTATGGTTCATCCTAGCTACTATCAAATGGGCATTA

CACATTATTAAGAGCCGATTGGTTGAATGGGAAAGTTCATCATGCATCCTCTTCCTTTGTTGCTGCTTCC

TCTTGTGCTGTTGGTGCTGTTGCCGTTCTTCCTTTTTAGATCCTTGTCTCGCCCTTGTTATCTCACATTG

ATTGTGCCCACTCCATCATTTTGGTCTTCCAGGCTTCGTTGTCAAATGCATCCACACCATGGCCACCGAG

GTCAAGATCGTCAAGCGTCAAATCTTTCTCCTCAGGCACCGGTTCATCAAATCCCACTACATGATTCAGT

TATGTAGAATGCAACATGCAAGAACAAGCTTAACCTGGGTGTGGTAAGGATGGAGTGGCTTTTGATCCAG

GATCTTGAACCTGTTCTTCACAGCTCCAAATGCCGTCTCAATGGTTACTCTAGGCTGAAGTGTGTGAGAT TGAACAACTCCCGTGGAGTCCTAGGGTAGTTCCTATGAGAGAACTCGTTAAGATGTGAAAGTGCGTTATA

TCGACTAGAGGGGGGGGGGGGGTGAATATGAGATTTTTAGAATTTCATCACTGAGGAAATTCCTTTTGAG

GAAATTCCTCACTGATGACTAACTTACAGCGGAAACAGTAAATGATCAGAAGTGCAAAGTTTCACAACAA

CAGTTTTTCAGAGTGAAGAATGTGAAAACAGATTGCACAGTGAGCAGGCACGCAGAATACAGATGAGGAA

TAGCTAAGGTGAAGAATTTGAGGTTGAGGAAATTCAGAGAAAGTCTTCAGAAAATTCTTCAAACAGTCAC

AGTGAAAGTTATCAACACATAATACGGGGAAAGTAAAGAGTTGAGGAATTAGAACCCGTTTCTCAGTGAA

GACAGTCGTTGATGACCCAGTTTCAACTGCTGTGACAGTCGTACATCTGGTTTGGAGCGGCTTGGTATTG

AAACCAAAGGACACCCAGTCCCGGGACACACAGTCCCTACCGTATTCTCCTTGAGCTAAGGACACACAGT

CCTTGCCCAACACTAGTGGTAAGTCTTCAGGGCAGACTTTCAAACCCTCACAAACTTGGTCACCCGGCGA

TCCACAATTGACTGCTGGAAAGCTCTAGACCATGACGCCTAACCGTCTGGAGGATGCACAGTCCTCAAAG

GTAAAAAGCTTCAGTCCCACACAGGAACAACTTCTTCAGTGATGCTCAATCACTAGGTTTGGTTTGTGGT

TTCGGTGGGTGGTGTATTTCCTCACTGATGAATTACTCTCGAAGGCTCTAAGGAATTTGGGTTGCTCTTA

TGACAAGTGTCAGTTTCTAACGGAGCAGCCAACCAGCTAGTGGTTGTGGGGGGTGGCTATTTATAGCCTG

GGAGCATCCCGACATGATTTGACACTAATGCCCTTAAATAATATGACCGTTGGAGTGGATAAGACCAGTG

ACTTGGCGTGGCTATCGAAACGGTCGGAACCCTCAACTGTGAGAGTCCTCATGTCACTCATATTCCTCAC

TTGAGGCTTTTGGTAGGATTAGGCTTGGGTTGAGCATCATGAGGAAATTCATTCCATAGTGTAACTTCGA

CCCCCTTTAACAGTATGGTGTTCCTATTACTCGAATGCGAAGAAAGTAAAACAGAAAGTGTAAATCTTCA

TGCTTCAATTCTTCAGAGTGATTTTCTTCAGGAACCACCGGACTTCTCAATATCAGTATCTTCATGAGGA

ATATCAATTTTATCGCAGATTCCTCGCGATTCATTTCTTCAGCTTCAGACCAATTTCTTCAACTGAAGAC

ATATATTTTTAGGGGTCGATATTCTTCAAATATCTCAAACTCCTCAATGACTTATAGATCCTGTGTACAC

TCACAAACACATTAGATACTTAACCTATAAGTCTTCAAACCACCAAAATCACTAAGGGGCACTAGATGCA

CTTACAAGATGGTATCTGGTCTTCCTGAAGGGTGGAAGAACACCTGGTTTACATGCATAACCAACATCTC

CTAGATAGAACTTGCCATCAGGNATGTTGATGTTAGCATCATGTGCTGACCCTTCCGAGTGAGCCAACAA

ATATGTGAACTTCATATCAAAGTCAACGTCAACAAGCACATTCTGGCTTGTGTAGTGCTTCCTCCCCCTA

AGTGCCGCAGCTTGTGACCTCAACACTCTGGGAGTGACATGAGTACCATCTATTGCCCCAGTGCAGTCCT

GAAATGGCAATAGAAGATCGTGTTAGTGCTCACCTTGAACAAATCTAGCATATGAAGCAATGAACTACAT

ACTACATTGCCCACCTTGAAGTATGGATACCATCTTGTGCTAGTACGAATCTTGCTAGGAGTCCGTCCAG

ATGGTGACTTGATCATTTCTCCTCTCAGCTCCCCAACAACATACAACACTTGCTTGAAGTATCTTGAGAT

GGTCTCCATTGATCTCCTGAACATGTTGTGGATAACCCCGAATCTGTGATTATGGCCAACAACATGAAGG

AGCATGGCCATTTACTCTTCTACACTGGTGTGGATGCTATCTTCTAGCAGCCTCCTGTTCCTCAAGATCT

GGACAAGTCTGTAAAATGGTGCTCTTTTCATCCGAAGCATCCACATGGCCCCTGTGTCGTTGCAGTTGTA

TATGTAGTTCAAATTGGCGATCCTCTCCTGCTCCCGCATAAGCATTAGAGCATATGTGATGATAGGTGTC

TCACCACGGTGAACAACTCTCTTGTGCATGAACATGACCCATGCCTGAATCACATATATCAGCGCTGCTG

ACTGAACTACCAGCCGCATCCGTGCGTCCATAACCTAGTCCACATCGAGGGTGCGTTGAGCAATGGGGAA

ATCGATCCTACACGCCTAACGGCCTAACAACGGAACCTAACATTAGGGACGGGGGACGGGGGGGGAGGGG

TGTTGCTTACCAGCATCAGAGACGAGAACAATGAAGGGGGGCCATGGCTGAGTCAAGGACGAGCAGCAAC

ACCTTGTGCCGTCGGATCTGCCGCCGTCACCGCCGCTTGCACAGAGTCGCCGCCCGTAGGTGGTGGTGAT

GCTAGAGGAAGAGGATGTCCTAGAGGTAGCGGTGATCGAGTAAATGGGTTGGGGAAGGGGGGGGGGGTGA

TACTATATTTGGCACCCTGTGGCCGCACCCGATTTCCATCTCCCGCCATCCCCACCCGCTTTCGTCTTGC

CCCCCGACGTGCGAGCTTGCACCGTCTTATGGATGGCTTGCTACAAACTGCCGATTCGGCAATGTCTAGC

GAGCCAGCCTTAGGGATGCTTTAAGCTTCATGCTGCTGTGAGCCAGCCGTGTGCGTTCTCAACCAAACGC

ACATTTGGCCCAACTTGGCTCTGATTGGGCCGGATGCTGGCAACCAAACGCGCCTTATACTTTCAAGTGC

TCCCACACGACGTTGCCCTTTGTATATAATCTAGGTCGAGGTCGAGTAGTTCCCATGTGGCGTATGGCTA

TATACCATGGAGAGTGCCGCTGTGACATCCCCGGGTATTGCTACAGTAACCCCTATAACCAGGCTAACGT

GATTACCGTGTTTAAGCTAATCACGTTTCTAAACAATGCTTGACCACTATGAATTTCATATACCTTTTCA

AATTCCACCACCTAAAGCCATTTAATTTTATTGTGACAAAATAAAACCTCAAACAGCAAATGGAAAATAT

TGCACACTTGTCAAAAATTCCAAAACAATTATTGATAAGGAAAACAACATCAACTTAATGTTGGTGTGAA

CCCCACCATCCAAAACGAAGCCTAACTGCAATTTAAAATGCACCTTTAAGTTTATGAAAAAATATAACAG

ATTCGAATGGGTTCCAAACTTTTTGTGGCATGACAGAATATTGCGTTATGATTGGTCGACCAAGTCATAC

ATTTTTTTGTACAACTTGTTGTTACAAAAATAAATAAAATAGAACTATAAAAGAAAGCACAAATGGAAAA

GGGAATAAAAGGAAAAGACTTACTGTGTATTGAGGCCCAAGTGCTACAGGCTAGCCCGACCCATTTAGGC

CAGCCTTCCCCTTCCCCTAGCCAACGACAGGAGAAGGTTTGCGTGCCATGGCTGCCGACGCTCGCGCGCG

GCCTTCGAGCCCCCGGGAGGATAAGAACGAGCCCCCTCGCTCTCTCTTGCTCCCGCAAACCCTAGCCCCC

TCCTGCACTCCCTCTCCTCATGAGTTCCCCCTTCCTCACGCCCGGCTGAGCGCAGCCATGGTTGCGCCAA

GCTAAGCTCGCGACCACCATCCTCCCTCCACTCCGACTAGCCATGGAGCTCCGCCTCGCCTTCCTCG

ACCCACTTGAGCGCTGGATCGAGCCGGAGCCAGACGGTAGTGAAAGTGCGTTATATCGACTAGAGGGGGG

GGGTGAATAGGAGATTTTTAGAATTTCATCACTGAGGAAATTCCTTTTGAGGAAATTCCTCACTGATGAC

TAACTTACAGCGGAAACAGTAAAGGATCAGGAGTGCAAAGTTTCCCTACATCAGTTTTTCAGTATGAGGA

ATGTGAAAACAGATTGCACAGTAAGCAGGCACGCAGAAAACATATGAGGATTAACTTGCGTGAAGAATTT

GGGGTTGAGGAATTTCAGAGAAAATCTTCAGCAAATTCTTCAAACAGTGACAGTGAAAGTCATCAACACA

TAATCTGAGGAATATAAAGAGTTGAGGAATTAGAACCCGTTTCACAGCGAAGACAGTAGTTGATGACCTA

GTTCCAACTGCTGTGACAGTTGTACATCTGGTTTGGAGCGGCTTGGTATTGAAACCAAAAGACACACAGT

CCCGGGACACACAGTCCCGGGACACACAGTCCCTACCGTATTCTCCTTGGGCTAAGAACACACAGTCCTC

GCCCAACACCCGTGGTAAGTCTTCAGGGCAGACTTTCAAACCCTCACAAACTTGGTCACCCGGCGATCCA

TAATTGACTGCTGGATTGCTCTAGACCATGACGCCTAACCGTCTGGAGGATGCACAATCCTCAAAGGTAA

AAGGCTTCAGTCCCACACAGGAACAACTTCTTCAGTGATGCTCAATCACTTGGTTCTCGGTTTGTGATTT

GGTGTTTGGGGTATTTTCTCACTGATGAATTACTCTTGAAGACTCTGAGGAATTGGGTTGCTCTTATGAC

AAGTGTCAGTTTCTAACGGAGCAGCCAACCAACTAATGGTTGTGGGGGACGGCTATTTATAGCCTGGGAA

CATCCCGACATGATTTGACACTAATGCCCTTAAATAATATGACCGTTCGTGTGGATAAGACCAATGATGT

GGCGTGACTATGGAAACGGTCGGAACCCTCAAATGTGAGAGTCCTCATGTCACTCATATTCCTCACTTGA

GGCTTTTGGTAGGATTAGGCTTGGGTTGAGCATCATGAGGAAATTCATTCCAAAGTGTAACTTCGACCCC

CTTTAACAATACGGTGTTCCTATTACTCAAGTGTGAAGAACACAAAACAGAAAGAGTAAATCTTCATGCT

TTAAAGTCTCCAGAATGATTTTCTTCAGGACACACCGAATTC
